# The association between hypertension and nonalcoholic fatty liver disease (NAFLD): literature evidence and systems biology analysis

**DOI:** 10.1080/21655979.2021.1933302

**Published:** 2021-06-06

**Authors:** Chongyang Ma, Kai Yan, Zisong Wang, Qiuyun Zhang, Lianyin Gao, Tian Xu, Jiayang Sai, Fafeng Cheng, Yuqiong Du

**Affiliations:** a School of Traditional Chinese Medicine, Capital Medical University, Beijing, China; b Department of Traditional Chinese Medicine, Beijing Chaoyang Hospital, Capital Medical University, Beijing, China; c School of Traditional Chinese Medicine, Beijing University of Chinese Medicine, Beijing, China; d Department of Oncology, The Third Affiliated Hospital, Beijing University of Chinese Medicine, Beijing, China

**Keywords:** Hypertension, NAFLD, systems biology, protein-protein interaction network, ALDH1A1

## Abstract

Nonalcoholic fatty liver disease (NAFLD) has become a major public health issue as its progression increases risks of multisystem morbidity and mortality. Recent evidence indicates a more complex relationship between hypertension and NAFLD than previously thought. In this study, a comprehensive literature search was used to gather information supporting the comorbidity phenomenon of hypertension and NAFLD. Then, systems biology approach was applied to identify the potential genes and mechanisms simultaneously associated with hypertension and NAFLD. With the help of protein-protein interaction network-based algorithm, we found that the distance between hypertension and NAFLD was much less than random ones. Sixty-four shared genes of hypertension and NAFLD modules were identified as core genes. Kyoto Encyclopedia of Genes and Genomes(KEGG) enrichment analysis indicated that some inflammatory, metabolic and endocrine signals were related to the potential biological functions of core genes. More importantly, drugs used to treat cardiovascular diseases, hypertension, hyperlipidemia, inflammatory diseases and depression could be potential therapeutics against hypertension-NAFLD co-occurrence. After analyzing public OMICs data, ALDH1A1 was identified as a potential therapeutic target, without being affected by reverse causality. These findings give a clue for the potential mechanisms of comorbidity of hypertension and NAFLD and highlight the multiple target-therapeutic strategy of NAFLD for future clinical research.

## Graphical Abstract

**Figure:**
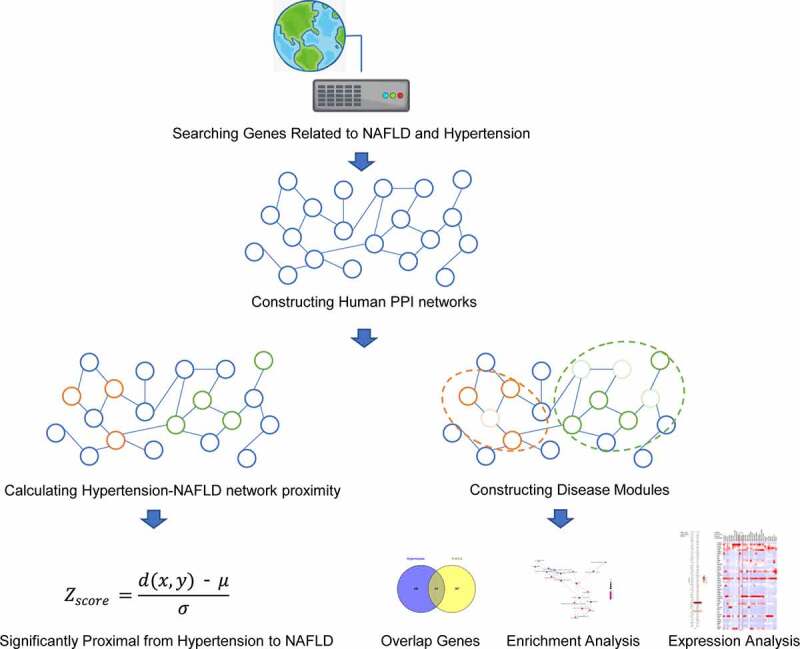

## Introduction

Nonalcoholic fatty liver disease (NAFLD) has emerged as the most common chronic liver disease worldwide and is becoming a major public health issue in China. According to previous reports, the prevalence of NAFLD in the Chinese population has increased to more than twice the rate in Western countries within a decade [1]. More importantly, nonalcoholic steatohepatitis (NASH) and hepatic fibrosis, as the two severe and progressive clinical forms of NAFLD, may ultimately lead to liver cirrhosis and hepatocellular carcinoma, as well as liver-related morbidity and mortality [2,3].

It is well accepted that NAFLD is a complex and systemic disorder with variations in genetic background, metabolic characteristics, cultural and socioeconomic factors, dietary habits, life styles as well as environmental risks, which could all contribute to NAFLD progression [4]. Importantly, NAFLD maintains a close association with obesity, hypertension, type 2 diabetes and other metabolic syndrome-related extrahepatic complications, which highlight the importance of the metabolic risk factors in NAFLD and promote a name change from NAFLD to metabolic associated fatty liver disease (MAFLD) [5]. As a core member of metabolic syndrome, hypertension is believed to have a strong link with NAFLD, and is supported by comprehensive evidences: 1) epidemiological investigation shows an approximately 49.5% NAFLD prevalence in hypertension patients, which is higher than the prevalence in the general population [6]; 2) NAFLD is associated with incident hypertension and endothelial dysfunction [7,8] and seems to be an independent risk factor of prehypertension and hypertension [9]; 3) accumulating evidence has demonstrated the existence of pathophysiological mechanisms including inflammation, renin-angiotensin system-sympathetic nervous system activation and insulin resistance in both hypertension and NAFLD [10,11]. Taken together, this comorbidity may in some ways lead to the complexity of NAFLD. Understanding shared genes and biological mechanisms of hypertension and NAFLD may help developing combined preventive strategies, exploring novel therapeutic approaches against NAFLD and contribute to making the most appropriate treatment plan for patients with this comorbidity.

Unfortunately, shared genes and mechanisms between NAFLD and hypertension are still poorly understood. Recently, bioinformatics scientists started to use biological networks as powerful resources for understanding the mechanisms in human complex diseases, for biological components such as genes and proteins always act via molecular interaction networks to generate, enhance or alter the disease phenotype [12]. These network-based systems biological approaches help uncover the molecular basis of the associations between diseases.

In this study, we hypothesized that NAFLD and hypertension shared common pathogenic mechanisms and similar underlying signaling pathways. To understand the relationship between hypertension and NAFLD, a literature search and system biology-based method were used.

## Methods

### Searching clinical studies for the associations between NAFLD and hypertension

A literature search was conducted in PubMed to identify relevant articles published on 31 March 2020. The search terms hypertension, nonalcoholic fatty liver disease, nonalcoholic steatohepatitis, liver fibrosis and combinations of them were used to identify articles. The search work was carried out by two authors independently. Only articles in the English language were included.

### Searching known genes associated with NAFLD, NAFLD-related phenotypes and hypertension

The list of genes for each phenotype of target diseases (hypertension, NAFLD, fibrosis and inflammation) was generated by searching two newly updated comprehensive discovery platforms designed to address the relationship between genes and human diseases: Disgenet (http://www.disgenet.org/)^13^ and CTD (http://ctdbase.org/) [14] database. The following keywords were used: hypertension, nonalcoholic fatty liver disease, inflammation and fibrosis. For CTD database, only marker/mechanism/therapeutic were used and for Disgenet database, genes from literature and animal models were excluded. These genes were identified by Entrez ID for further analysis. To explain the interactions between the genes of each disease and phenotypes, Venny’s online software (http://bioinfogp.cnb.csic.es/tools/venny/index.html) was used for overlapping analysis. Search Tool for the Retrieval of Interacting Genes database website (STRING: http://string.embl.de/) was used to construct an interaction network of overlapped genes [15].

### Human genomic protein-protein interaction (PPI) networks

The human protein-protein interaction (PPI) network was constructed from Professor Barabasi’s team [16], which was integrated with 15 commonly used protein-protein interactome databases and in-house data. Inferred data including evolutionary analysis, gene expression data and metabolic associations are excluded and a network containing 16,677 nodes (by Entrez gene ID) and 243,603 edges is obtained.

### Calculation of network proximity

To quantitate whether there is any statistical influence of hypertension on NAFLD, an efficient way to capture network proximity between one disease to another disease is by the z-score, which relies on the closest distance measured by the average shortest path length *d*(*x*, *y*) between hypertension genes (*x*) and NAFLD genes (*y)*. The *Z*_*score*_ is obtained by comparing the observed closest distance to a reference distance distribution between a randomly selected group of genes of matching size and degree distribution as the seed disease genes and target disease genes in the above human interactome. According to the standard normal distribution, *Z*_*score*_ ≤ −1.645 (one-sided P value < 0.05) was considered as significantly proximal from one disease to another disease [17].

$$Z_{score}=\frac{d\left(x,y\right)-\mu }{\sigma }$$

### Disease module construction

According to previous study, complex disease genes rarely resulted by the abnormality in a single gene, and the specific disease modules could be identified via PPI network-based systems biology analysis. In this study, the hypertension and NAFLD disease modules are built through the DIAMOnD algorithm [18], which is based on an iterative scheme that exploits the network’s topology. After obtaining genes involved in hypertension module and NAFLD module, overlap genes between the genes are identified as core genes between hypertension and NAFLD.

### Enrichment analysis

Metascape (http://www.metascape.org) is used for automated meta-analysis to understand common and unique pathways in NAFLD, NAFLD-related phenotype and hypertension [19]. Gene Ontology (GO) enrichment of these genes includes biological process (BP), molecular function (MF) and cellular component (CC). Only terms with P-value < 0.01, minimum count of 3 and enrichment factor of >1.5 were considered as significant. For a comparison between hypertension module and NAFLD module, the ClusterProfiler package of R 3.5.0 software is adopted to conduct GO enrichment [20]. Kyoto Encyclopedia of Genes and Genomes (KEGG) pathway enrichment analysis of core targets is also carried out and visualized using this package. P-value < 0.05 was set to be significant as previously studied [21].

### The drug–gene interaction network

DGIdb (version 4.0) is an open-source project that help users mine existing resources and generate assumptions about how genes are therapeutically targeted or prioritized for drug development [22]. All the core genes were used for drug identification and the parameters were set as: preset filters: FDA approved; advanced filters: disease agnostic sources and all the default. Cytoscape software (version 3.2.1) was used for drug-gene interaction network construction.

### Expression of core genes

Analysis of gene (mRNA) expression levels in the liver tissue was conducted by retrieving data from The Genotype-Tissue Expression (GTEx) Consortium (Version 7) [23]. Global protein expression levels were explored using the FunRich software based on the information retrieved from the UniProt database [24].

### Expression of ALDH1A1 gene in hypertensive liver

We searched the GEO database and obtained a genetic dataset (GSE19817). This dataset contained 67 samples from male mice with different levels of blood pressure (High, normal and low). GEO2R, a useful online tool for identifying DEGs in the GEO platform, was used to identify DEGs between hypertensive liver tissues and normal blood pressure liver tissues. |log2FC| > 0.3 and adj. P-value < 0.05 (Fold change = hypertensive sample expression/normal sample expression) were set as the cutoff standard.

In this article, to understand the relationship between hypertension and NAFLD, we first searched the PubMed database to gather clinical studies that supported a strong relationship between hypertension and NAFLD. Then we applied a protein-protein interaction (PPI) network-based systems biology approach to evaluate the significance of the network distance between hypertension and NAFLD. Finally, we identified some commonly shared genes and biological mechanisms of NAFLD and hypertension disease modules, which indicate that multiple-target therapeutic strategy could be more appropriate for future NAFLD treatments.

## Results

### Epidemiological basis linking NAFLD and hypertension

We identified 26 studies published in the last ten years reporting either that hypertension may contribute independently to the development of NAFLD or NAFLD was considered as a risk factor of hypertension. Fourteen studies showed that hypertension is a risk factor for NAFLD, ten studies identified NAFLD as a risk factor for hypertension, and two studies reported a bidirectional relationship (Supplementary Table 1). All these new lines of evidence indicated a potential reciprocal causation of NAFLD and hypertension, and implied the existence of a complex and intertwined biological basis linking blood pressure regulation to metabolic and histologic conditions in the liver.

### Genes associated with NAFLD, NAFLD-related phenotypes and hypertension

**Figure 1.**
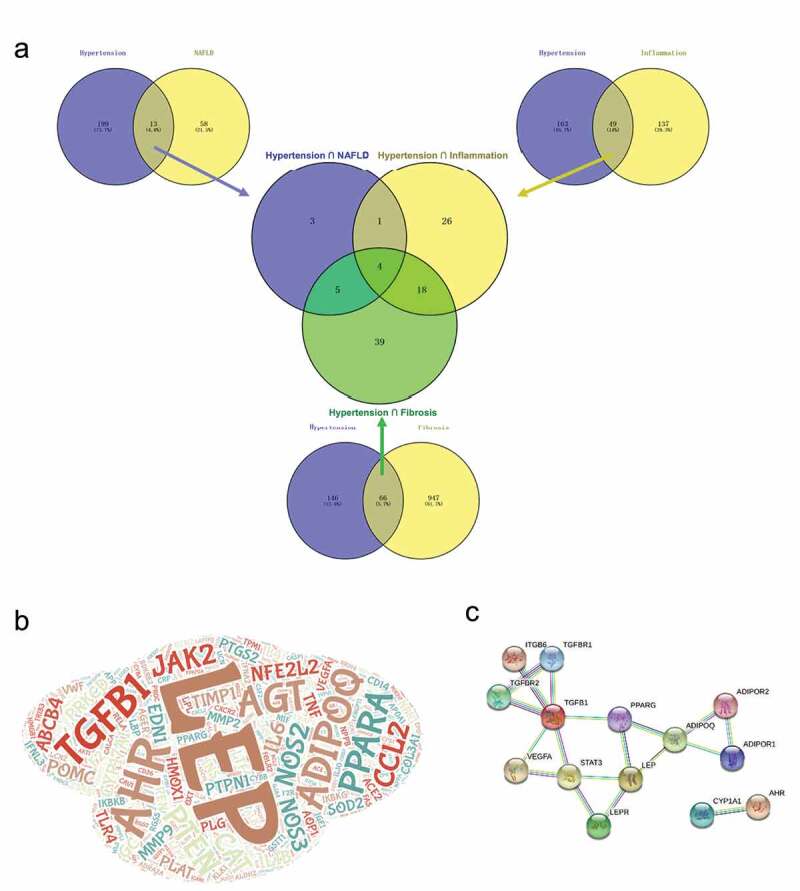
Common genes among Non‐alcoholic fatty liver disease (NAFLD), NAFLD-related phenotypes and hypertension. (a) Venn diagram showing the number of genes that are shared by NAFLD, NAFLD-related phenotypes and hypertension. (b) Word cloud diagram showing the genes in the NAFLD, NAFLD-related phenotypes and hypertension. The size of word depends on term frequency in the four gene list. C. Protein-protein interaction network of intersective genes among NAFLD, NAFLD-related phenotypes and hypertension

Although the clinical phenomenon of comorbidity of NAFLD and hypertension has been known for a long time, the genes potentially involved in both diseases or related pathophysiological processes were studied only in a few existing works. Through database searching, we obtained 1292 unduplicated genes associated with NAFLD, NAFLD-related phenotypes and hypertension. The number of genes used for further analyses were as follows: 71 genes associated with NAFLD, 212 genes with hypertension, 421 with inflammation and 1013 genes with fibrosis(Supplementary Table 2). We used a web-based tool to compare overlapping genes between diseases with related phenotypes. As is shown in Figures 1A, 13 genes were shared by NAFLD and hypertension, which accounted for 18.31% of NAFLD genes and 6.13% of hypertension genes. Forty nine genes were shared by inflammation and hypertension, and 66 genes were shared by fibrosis and hypertension. This intersection analysis showed that the final list of shared genes among hypertension, NAFLD, fibrosis and inflammation contained only four genes, including LEP, ADIPOQ, AHR and TGFB1 (Figure 1B), which could be regarded as important genes related to hypertension and NAFLD. Using STRING database, we constructed a protein-protein interaction network containing 14 genes for NAFLD-hypertension correlation (Figure 1C).

### Gene ontology terms enriched in genes of NAFLD, NAFLD-associated phenotypes and hypertension

**Figure 2.**
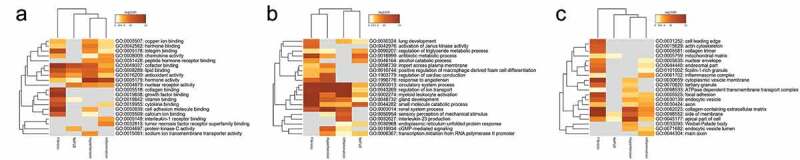
Gene Ontology (GO) terms of genes among Non‐alcoholic fatty liver disease (NAFLD), NAFLD-related phenotypes and hypertension. Heat map showing biological process (a), molecular function (b) and cellular component (c) that are shared by NAFLD, NAFLD-related phenotypes and hypertension

To identify potential biological mechanisms related to NAFLD, NAFLD-associated phenotypes and hypertension, we first identified all statistically enriched terms categorized by biological processes, molecular functions and cellular components, and then calculated accumulative hypergeometric P-values and enrichment factors for further filtering, as mentioned in the methods part. The overrepresented biological processes molecular functions and cellular components are summarized in Figure 2A, B and C. Cofactor binding, lipid binding, antioxidant activity and hormone activity were associated with all these diseases and phenotypes. Molecular function enrichment indicated that circulatory system process, regulation of ion transport, myeloid leukocyte activation, gland development and small molecule catabolic process. Cellular components enrichment indicated genes in all these diseases and phenotypes were significantly enriched in side of membrane and apical part of cell.

### Protein-protein interaction network-based proximity between genes of hypertension and NAFLD

To understand the relationship of hypertension and NAFLD, we used a network-based proximity measure to quantify the interplay between hypertension and NAFLD (Supplementary Figure 1). After calculating average shortest path length between hypertension genes (*x*) and NAFLD genes and average shortest path length between random control genes, *Z*_*score*_ of hypertension and NAFLD was −8.84 (p < 0.05).

### Hypertension and NAFLD modules identification

**Figure 3.**
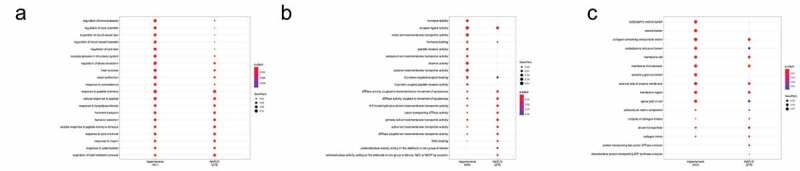
Gene Ontology (GO) terms of genes among Non‐alcoholic fatty liver disease (NAFLD) module and hypertension module. Bubble plots showing biological process (a), molecular function (b) and cellular component (c) that are shared by NAFLD module and hypertension module

Although network-based proximity offers information on the similarity of hypertension and NAFLD, it does not help in identifying core genes that influence both diseases. And above disease-associated genes search tends to obtain an incomplete landscape of diseases, which limited the number of overlapped genes. Therefore, a network-based disease module algorithm was used. After employing DIAMOnD algorithm, 512 and 371 genes were obtained associated with hypertension and NAFLD, respectively. As shown in Figure 3, GO enrichment was used to compare the common terms of these two modules. Some biological progresses related to cardiovascular system were obtained, including regulation of blood pressure, regulation of blood vessel size, regulation of blood circulation, heart process and heart contraction. Response to LPS was also enriched, indicating immune system may be involved in the interaction of hypertension and NAFLD. Some metabolic processes were obtained such as response to acid chemical, response to insulin, response to carbohydrate and regulation of lipid metabolic process. Endocrine system seems to be also involved, including response to corticosteroid, response to peptide hormone, hormone transport and hormone secretion. Molecular functions enrichment indicated G protein-coupled receptor binding, ATPase activity and NAD binding. Cellular components enrichment indicated that these genes are localized in membrane region, endoplasmic reticulum lumen, collagen trimer, extracellular matrix component and blood microparticle.

### Core genes of hypertension and NAFLD modules and enrichment analysis

**Figure 4.**
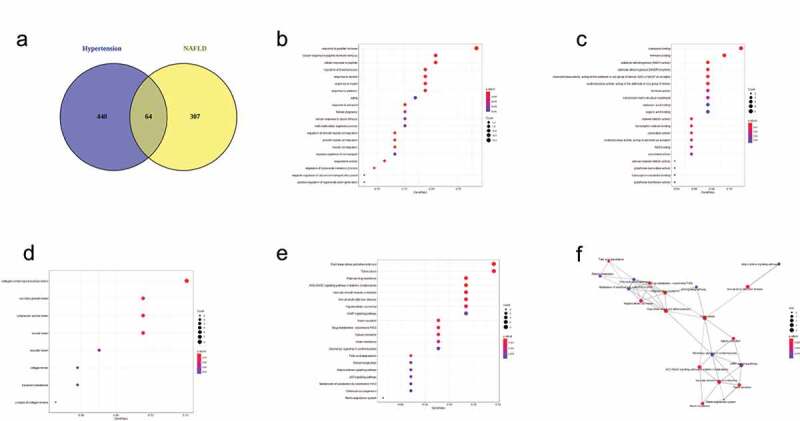
Gene Ontology (GO) and KEGG enrichment of 64 shared genes of Non‐alcoholic fatty liver disease (NAFLD) module and hypertension module. (a) Venn diagram showing the number of genes that are shared by NAFLD module and hypertension module. Bubble plots showing biological process (b), molecular function (c), cellular component (d) and KEGG pathway (e) terms of 64 shared genes of NAFLD module and hypertension module

As shown in Figure 4A, 64 unduplicated genes were recognized as core genes of hypertension and NAFLD modules and used for enrichment analysis (Supplementary Table 3). BP enrichment (Figure 4B) indicated that these core genes related to regulation of blood pressure, response to alcohol, insulin, antibiotic and estradiol and regulation of superoxide metabolic progress. Some oxidative stress-related MFs were obtained including aldehyde dehydrogenase (NAD+) activity, oxidoreductase activity, peroxidase activity, NAD binding, antioxidant activity (Figure 4C). CC enrichment indicated core genes were localized in collagen-containing extracellular matrix, collagen trimer and basement membrane (Figure 4E). KEGG enrichment indicated core genes are enriched in fluid shear stress and atherosclerosis, AGE-RAGE signaling pathway in diabetic complications, renin secretion, vascular smooth muscle contraction, nonalcoholic fatty liver disease, fatty acid degradation, hepatocellular carcinoma, Insulin resistance, adipocytokine signaling pathway, renin-angiotensin system, retinol metabolism and p53 signaling pathway (Figure 4F).

### Hypertension and NAFLD drug-gene interaction network

**Figure 5.**
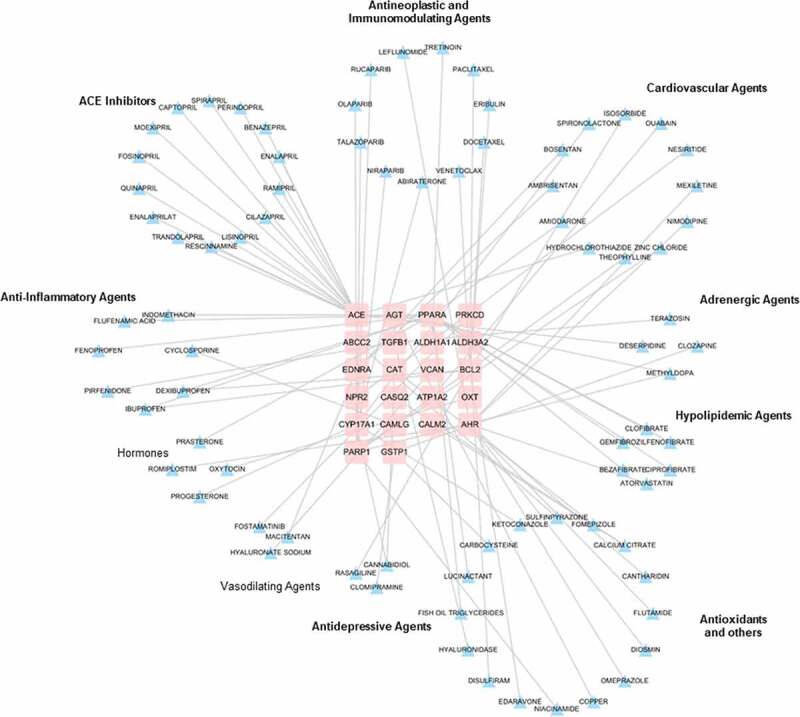
Non‐alcoholic fatty liver disease (NAFLD) and hypertension: gene-drug interaction network

To discover the potential therapeutic drugs, a drug-gene interaction network for hypertension and NAFLD was constructed using 64 shared genes above. Through DGIdb database, only 22 genes were considered as therapy target genes with FDA-approved drugs, indicating that plenty of work needs to be done in drug discovery based on these identified genes. In the drug-gene network, all the FDA drugs are currently in use for treating cardiovascular diseases, hypertension, hyperlipidemia, inflammatory diseases and depression (Figure 5). These results highlighted the possibility and requirement of drug repositions for treating NAFLD.

### Expression patterns of shared genes

**Figure 6.**
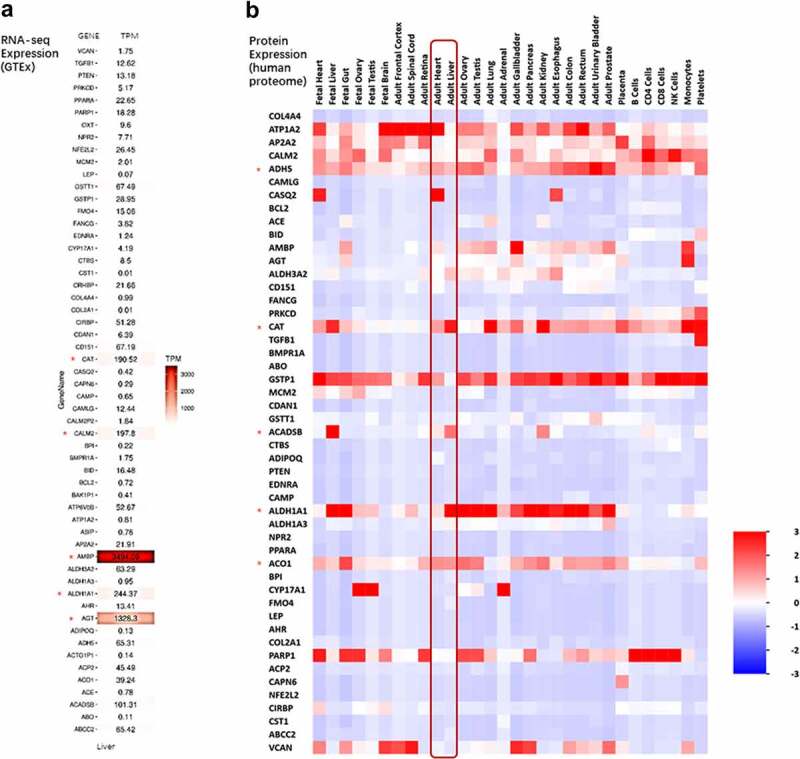
Expression pattern in different tissue of 64 shared genes. (a) MRNA expression heat map for the list of the 64 shared genes in liver. Expressive data was extracted from The GTEx Consortium. (b) Protein expression heat map for the list of the 64 shared genes in different tissues. Expressive data was extracted from UniProt database by FunRich tool

To understand the potential biological function, we integrated the list of 64 core genes with public OMICs data, including transcriptomics and proteomics. We constructed a heat map based on the lise of 64 genes (input gene list), in which liver expression (RNA-seq expression) levels generated using data extracted from The GTEx Consortium (Figure 6A) were shown. Genes like Alpha-1-Microglobulin/Bikunin Precursor (AMBP), angiotensinogen (AGT), Aldehyde Dehydrogenase 1 Family Member A1 (ALDH1A1), Calmodulin 2 (CALM2) and Catalase (CAT) were found highly expressed in the liver. Conversely, some transcripts had very low expression levels in normal liver, such as Leptin (LEP), Angiotensin I Converting Enzyme (ACE), BCL2 Apoptosis Regulator (BCL2) Adiponectin, C1Q And Collagen Domain Containing (ADIPOQ) and Transforming Growth Factor Beta 1 (TGFB1). Protein expression results of 64 genes in different tissues based on the whole proteome data indicated that most of these genes had similar levels of expression in the liver and heart. And we identified some high expressed genes in the liver, including ATPase Na+/K+ Transporting Subunit Alpha 2(ATP1A2), Formaldehyde Dehydrogenase (ADH5), Catalase (CAT), Acyl-CoA Dehydrogenase Short/Branched Chain (ACADSB), Aldehyde Dehydrogenase 1 Family Member A1 (ALDH1A1) and Aconitase 1 (ACO1). These data indicated above identified genes may involve both of liver physiology and hypertension-NAFLD pathology, which could be considered as potential therapeutic druggable targets.

### ALDH1A1 expression level validation

We focused on the expression levels of the ALDH1A1 genes in the GSE19817 datasets. The results showed that the expression level of ALDH1A1 was statistically significantly elevated in hypertensive liver tissues compared with their expression in normal blood pressure liver tissues (Supplementary Figure 2).

## Discussion

Traditionally speaking, NAFLD was considered as simple ‘hepatic manifestation’ of MetS for its strong association with other components of MetS. Recently, researchers started to think over the role of NAFLD in the future development of other MetS components and the role of MetS components in the development and progression of NAFLD, which may have closer mutual interrelationships and limit NAFLD therapeutic developments [11]. Here we focused a close relationship between hypertension and NAFLD. Recent clinical evidence showed that the prevalence of hypertension was higher in patients with NAFLD or NASH than in the general population (39.3% and 68.0%, vs 29.0%) [25]. A meta-analysis of related cross-sectional studies showed that pooled odds ratio for prevalent hypertension of NAFLD subjects versus non-NAFLD subjects was 1.24 (95% CI: 1.14–1.36), without any potential heterogeneity among the individual studies [26]. On the other hand, up to 57.5% of hypertensive patients had NAFLD [27], which was much higher than in the general population. According to another meta-analysis published in 2016, the comorbidity of NAFLD and hypertension was reported in the majority of the included studies, while the comorbidity of NASH and hypertension were reported in only four studies [28]. The pooled overall prevalence of hypertension estimated in NAFLD and NASH patients was 39.34% and 67.97%. Another meta-analysis containing 411 biopsy-proven NAFLD patients reported an odds ratio of 1.94 (95% confidence interval 1.00 to 3.74) for liver progressive fibrosis in people with hypertension compared with those without hypertension [29]. Our literature searching approach identified 26 studies reporting either that hypertension may contribute independently to the development of NAFLD or NAFLD was considered as a risk factor of hypertension (Supplementary Table 1). Based on a newly published review, in the past ten years, several cross-sectional studies have indicated that the presence and severity of NAFLD are associated with the presence of both prehypertension and hypertension [9]. Several prospective cohort and cross-sectional studies indicated that hypertension was an independent predictor of NAFLD [30–35]. Interestingly, prehypertension was also associated with NAFLD, because the odd ratios increased in a specific range of blood pressure [36,37]. NASH and fibrosis, even HCC, were associated with hypertension. A recently published cross-sectional study indicated that a history of hypertension was associated with a higher probability of NASH [38]. These clinical data indicated hypertension-induced NASH, which could be progressed from nonalcoholic fatty liver. More importantly, the drugs targeting

different mechanistic pathways treating NASH will become analogous to the treatment of hypertension [39]. For fibrosis, a prospective cohort study containing 271 Italian subjects with NAFLD reported that the diagnosis of hypertension (odds ratio = 4.8; P = 0.028) was considered as an independent predictive factor of worsening fibrosis over a mean follow-up of 6.4 years [40]. However, because of the unique features of the population, any independent association of BMI with the extent of baseline fibrosis might be neglected in analysis. A real-world cohort study in Germany found that hypertension was an independent predictor of advanced fibrosis [41]. In the elderly NAFLD patients, hypertension was also associated with advanced fibrosis [42]. A retrospective cohort study of 271,906 patients with NAFLD showed that the risk of progression from cirrhosis to HCC was 3.5-fold higher in patients with hypertension than those free of hypertension [43], which was confirmed by another case-control study [44]. Interestingly, hypertension was not associated with the risk of HCC progression in the absence of cirrhosis. On the basis of these phenomena, future studies should pay attention to those patients with NAFLD/NASH and hypertension as screening for HCC. However, it is still unclear if management of hypertension would reduce HCC risk. Some prospective cohort studies including subjects with different clinical conditions showed that NAFLD was an independent risk factor for hypertension [45–51]. A prospective cohort study including 11,350 subjects without prehypertension in 5 years follow-up showed that NAFLD was an independent risk factor of prehypertension, because the severity of NAFLD was positively associated with the risk of prehypertension [52]. Other studies showed that lean-NAFLD and a high risk of advanced fibrosis also increased the incidence of hypertension [53,54]. Two prospective cohort studies supported the existence of a bidirectional relationship between NAFLD and hypertension, and the latter is strongly associated with morbidity and mortality of cerebrovascular disease and cardiac disease [55,56]. Therefore, recognizing the biological molecular basis could help identifying novel drugs treating NAFLD and developing potential strategies.

In the present systems biology analysis, some common BPs and MFs related to confirmed gene clusters of hypertension, NAFLD and NAFLD phenotypes included lipid binding, antioxidant activity, hormone activity, circulatory system process, myeloid leukocyte activation and small molecule catabolic process, which are involved in cardiovascular system, metabolic system, immune system and endocrine system. Of note, experimental evidence confirmed that some immune effector cells, including natural killer cells, macrophages, T cells, and B cells, promoted the progression in patients with NAFLD to steatosis, cirrhosis and hepatocellular carcinoma [57–59]. We identified that leukocytes had a strong association with NAFLD, NAFLD-related phenotypes and hypertension, which could be considered as an important immune cell type connecting hypertension and NAFLD. Pathologically, the progression of NAFLD in steatohepatitis was characterized by hepatic leukocyte accumulation, which means that leukocytes were recruited from the blood and relocated at the liver tissue. Experiments showed that recruitment of leukocytes contributed to the development of fibrosis, thus targeting molecules involved in this progress had the therapeutic potential for NAFLD [60]. However, another study disagreed with the cellular crosstalk between leukocytes and matrix and put forward that other cells in the liver may have a pivotal role in both fibrogenesis and liver repair [61]. Similarly, the recruitment of leukocytes to the vascular wall is also a key step in hypertension development [62].

To evaluate the possibility of hypertension-NAFLD interaction, we calculated the network proximity between hypertension genes and NAFLD genes in a human PPI interactome and compared it with groups of random selected genes. These data showed hypertension genes were more adjacent to NAFLD genes than random genes in the PPI network, indicating a strong association between these two diseases. Preclinical evidence showed that, compared to a normal chow diet, moderate high-fat diet improved blood pressures of maternal mice and their offspring. Importantly, the probability of offspring hypertension was particularly increased, if other factors remain unaltered [63]. Another study indicated that hypertension precipitated hepatic steatosis and induced severe liver fibrosis through oxidative stress after a 20-weeks feeding of a choline-deficient diet, without any changes on body weight [64]. Chronic high fructose intake increased liver fat deposition and fibrosis in hypertensive rats rather than non-hypertensive rats [65]. Some anti-hypertension drugs were found to provide a beneficial modulation against NALFD and NAFLD-associated phenotypes, including inflammation and fibrosis [66–70]. However, the specific molecules involving in both diseases need to be identified. Based on disease-module theory, which indicated each disease can be linked to a well-defined local neighborhood of the interactome, we used a well-recognized algorithm on the same PPI interactome to identify potential modules related to hypertension and NAFLD. Overlapped genes of hypertension modules and NAFLD modules were five times than size of overlapped genes of hypertension and NAFLD, which indicated the application of disease-module theory is a well-performed method in understanding disease-disease interactions. Of note, some oxidative stress-related progresses were enriched by both diseases, indicating application of nicotinamide adenine dinucleotide (NAD+) against hypertension-NAFLD concurrence. Recent studies indicated that a decreased level of NAD+ in the liver, which is generated from nicotinamide, was followed by high-fat diet (HFD) feeding [71]. Supplementation of NAD+ precursors decreased HFD-induced hepatic lipid and fat mass accumulation, and improved insulin activity [72]. Our data indicated potential clinical usage of oxidative-related drugs such as edaravone against hypertension and NAFLD complex, which has been reported holding a liver protective effect [73].

The present overlap of genes of hypertension and NAFLD modules were enriched by the KEGG database. Insulin resistance and AGE-RAGE signaling pathway in diabetic complications signals were all enriched, indicating a complicated relationship between NAFLD, hypertension and diabetes. A consensus of international experts proposed that metabolic (dysfunction) associated fatty liver disease (MAFLD) was a more appropriate term than NAFLD, given the common association between NAFLD and metabolic dysfunction including hypertension, diabetes and obesity [74]. Renin-angiotensin system (RAS) was one of the most well-published mechanisms and therapeutic targets against both NAFLD and hypertension [75,76]. According to expression information derived from Gene cards database, both RNA-seq and microarray data indicated that RAS constituents, including a classical angiotensin-converting enzyme (ACE)/angiotensin II (AngII)/type 1 angiotensin receptor (AT1R) axis and a new angiotensin-converting enzyme 2 (ACE2)/angiotensin 1–7(Ang1-7)/Mas axis, were expressed in normal liver, heart and kidney. AngII promotes insulin resistance, de novo lipogenesis and pro-inflammatory cytokine production, and triggers liver inflammation and fibrogenesis [77], while active Ang (1–7) signal inhibits liver lipogenesis, fatty acid oxidation, inflammation and fibrosis [78,79]. These data were confirmed by molecular agents and gene manipulation models [80–82].

By analyzing transcriptomics and proteomics data, we identified aldehyde dehydrogenase RALDH1 (ALDH1A1, alternatively known as retinaldehyde dehydrogenase 1 or RALDH1) gene as a mediator related to both liver physiology and hypertension-NAFLD interaction, which has also been identified as differently expressed gene of a high blood pressure vs normal blood pressure mice gene series. ALDH1A1 is an important enzyme associated with metabolic diseases, which catalyzes the second and irreversible step of retinaldehyde oxidation to vitamin A (retinoic acid). In liver, ALDH1A1 has been reported to be present in rat hepatocytes, hepatic stellate cells (HSC) and Kupffer cells [83]. In hepatocytes, ALDH1A1 deficiency resulted in a decreased hepatic glucose production and repressed hepatic triglyceride production [84]. In Kuppfer cells, upregulating ALDH1A1 induced a high level of TNF signal activation [85]. It seems like inhibiting ALDH1A1 would have some benefits against hypertension-NAFLD complex. In the comparisons among the normal, steatosis and NASH patients, the expression of ALDH1A1 was significantly higher in the NASH livers than normal livers [86]. More interestingly, analysis of the Oncomine database showed that the expression of ALDH1A1 was significantly upregulated in the hepatocellular carcinoma (HCC) tissues than in the normal tissues [87]. Pre-clinical studies indicated that ALDH1A1 knockout mice and inhibitors treatment decreased adipose tissue weight, adipocyte size and fatty acid synthesis in the livers after high-fat diet [88,89]. Of note, it is well established that retinoic acid induced by ALDH1A1 activated transcriptional networks controlled by retinoic acid receptors (RARs) and retinoid X receptors (RXRs), while RAR-mediated signaling most consistently induced suppression of hepatic non-esterified free fatty acids (NEFAs) and TG accumulation and RXR-mediated signaling may cause opposing effects at different levels in the metabolic pathway, leading to hepatic lipid accumulation [90]. ALDH1A1 was considered to play an important role in the detoxification of lipid-derived aldehydes, including 4-hydroxy-2-nonenal and acrolein, compounds that mediate oxidative stress [91]. These studies could explain the contradiction between a better metabolic phenotype by diminishing retinoic acid synthesis and a beneficial effect of retinoic acid against NAFLD development. Further studies are needed to establish the specific function of ALDH1A1 in the progress of NAFLD.

In this study, an important limitation of our systems biology approach was the lack of experimental validation of the roles of the identified gene list on the cross-talk between NAFLD and hypertension. Since the biological mechanisms of NAFLD and hypertension comorbidity are poorly understood, evidence supported the associations of the above genes with NAFLD, and hypertension comorbidity is also limited. Experiments using a complex model of NAFLD and hypertension should be designed in the future to identify the role of these genes in molecular agents or genetic manipulation.

## Conclusions

In conclusion, clinical evidence indicated a bidirectional effect of hypertension and NAFLD. The common biological processes and shared genes identified by the present systems biology approach could explain multiple molecular bases among hypertension and NAFLD to some extent. Our results indicate that NAFLD has common disease pathogenic mechanisms with hypertension and could be considered as a systemic disease which requires multi-target therapeutic strategies rather than a single treatment.

## Supplementary Material

Supplemental Material

## Data Availability

The data that support the findings of this study are available with the corresponding author upon reasonable request.

## Highlights

1. NAFLD serves as an independent risk factor of hypertension, as well as hypertension is considered as an independent risk factor of NAFLD.

2. Sixty-four genes involved in the complex relationship between hypertension and NAFLD.

3. ALDH1A1 is identified as a potential therapeutic target treating the comorbidity of hypertension and NAFLD.
