# Supplementary material for: The association between hypertension and nonalcoholic fatty liver disease (NAFLD): literature evidence and systems biology analysis
Source: Bioengineered. 2021 Jun 6;12(1):2187–202. doi: 10.1080/21655979.2021.1933302 (PMC8806441; doi:10.1080/21655979.2021.1933302)
Supplement: Supplemental Material [file KBIE_A_1933302_SM4745.zip › supplementary/Stable.docx]

**Supplementary Figure 1. Protein-protein interaction network-based proximity between genes of hypertension and NAFLD.**

**Supplementary Figure 2. The expression level of Aldh1a1 in hypertensive liver tissues compared with their expression in normal blood pressure liver tissues**

Supplementary Table 1. Clinical evidence of NAFLD and hypertension

| References | Country | Type | NAFLD Diagnosis | Follow-up | Sample size | Key Results |
| --- | --- | --- | --- | --- | --- | --- |
| Sorrentino et al. 2010 | Italy | Prospective Cohort | Biopsy specimens | 6.4 years | 271 subjects with NAFLD | Hypertension was an independent predictor of worsening fibrosis: OR = 6.3 (95% CI: 4.1–14) |
| Tsuneto et al. 2010 | Japan | Prospective Cohort | Abdominal ultrasonography | 11.6 years | 1635 subjects without NAFLD | Hypertension was an independent predictor of NAFLD: RR = 1.31 (95% CI: 1.01–1.71) |
| Zhang et al. 2015 | China | Prospective Cohort | Abdominal ultrasonography | 3.27 years | 15791 subjects without NAFLD | Hypertension was an independent predictor of NAFLD: HR = 1.75 (95% CI: 1.51–2.02) |
| Wu et al. 2015 | China | Prospective Cohort | Abdominal ultrasonography | 2 years | 10915 subjects without NAFLD and with normal BP | Higher systolic blood pressure levels (120–129 mmHg) had a higher risk of NAFLD: HR=2.38 (95% CI: 1.75–3.23) |
| Aneni et al. 2015 | USA | Cross-sectional study | Abdominal ultrasonography | NA | 5362 subjects with different levels of blood pressure | Hypertension was an independent predictor of NAFLD: RR = 1.8 (95% CI: 1.4–2.3) |
| Wang et al. 2016 | China | Cross-sectional | Transient Elastography | NA | 836 subjects with different levels of blood pressure | The ORs for NAFLD increased progressively across the blood pressure categories. High normal blood pressure(SBP 130–139 mmHg and/or DBP 85–89 mmHg): 1.567 (95% CI: 1.041–2.356); Grade 1(SBP 140–159 mmHg and/or DBP 90–99 mmHg): OR = 2.430 (95% CI: 1.559–3.789); Grade 2 (SBP 160–179 mmHg and/or DBP 100–109 mmHg.): OR = 4.598 (95% CI: 2.158–9.793); Grade 3(SBP ≥180 mmHg and/or DBP≥110 mmHg): OR = 3.029 (95% CI: 0.631–14.549) |
| Petta et al. 2018 | Italy | Cross-section | FibroScan | NA | 890 subjects with and without NAFLD | Hypertension was an independent associated with NAFLD: OR =1.55 (95% CI: 1.09-2.21) |
| Labenz et al. 2018 | Germany | Real‐world cohort | Biopsy specimens | NA | 261 subjects with non‐cirrhotic NAFLD | Hypertension were independent predictors of advanced fibrosis: OR = 2.91 (95% CI: 1.12‐7.18) |
| Han et al. 2019 | China | Cross-section | Abdominal ultrasonography | NA | 6849 subjects with and without nonalcoholic fatty liver disease | Hypertension was an independent predictor of NAFLD: OR = 1.325 (95% CI: 1.157–1.518) |
| Gawrieh et al. 2019 | USA | Cross-section | Biopsy specimens | NA | 480 subjects with NAFLD | History of hypertension was associated with higher probability of NASH F2-3: OR=1.5 (95% CI: 0.9–2.5) |
| German et al. 2019 | USA | Case control | Biopsy specimens | NA | 102 subjects (34 HCC cases and 68 non-HCC controls) | Hypertension was associated with an increased risk of developing HCC: OR =5.80 (95% CI: 2.01-16.75) |
| Younossi et al. 2020 | USA | Cross-section | Fatty Liver Index | NA | 29822 subjects with NAFLD | Hypertension was an independent predictor of NAFLD: OR=1.83 (95% CI: 1.63-2.06) |
| Kanwal et al. 2020 | USA | Retrospective Cohort | Serum transaminase | 9 years | 271906 subjects with NAFLD | Hypertension was an independent predictor of incident cirrhosis or HCC: HR= 3.52 (95% CI: 3.34-3.71) |
| Pitisuttithum et al. 2020 | ASIA | Cross-section | Biopsy specimens | NA | 175 elderly subjects with NAFLD | Hypertension was associated with advanced fibrosis in elderly patients: OR=3.68 (95% CI: 1.11–12.23) |
| Lau et al. 2010 | Germany | Prospective Cohort | Abdominal ultrasonography and Serum transaminase | 5.3 years | 3191 subjects aged 20–79 years | NAFLD was associated with increased risk of incident hypertension: OR=3.1 (95% CI: 1.7–5.8) |
| Sung et al. 2014 | Korea | Prospective Cohort | Abdominal ultrasonography | 5 years | 11448 subjects without hypertension | NAFLD was associated with increased risk of incident hypertension: OR=1.36 (95% CI: 1.1–1.7) |
| Ryoo et al. 2014 | Korea | Prospective cohort | Abdominal ultrasonography | 5 years | 22090 subjects without hypertension | NAFLD was an independent risk factor for hypertension. Mild NAFLD: HR=1.07 (95% CI: 1.00–1.15); Moderate to severe NAFLD: HR=1.14 (95% CI: 1.00–1.3) |
| Ryoo et al. 2014 | Korea | Prospective cohort | Abdominal ultrasonography | 5 years | 11350 subjects without prehypertension | NAFLD was an independent risk factor for prehypertension. Mild NAFLD: HR=1.18 (95% CI: 1.07–1.31); Moderate to severe NAFLD: HR=1.62 (95% CI: 1.21–2.17) |
| Feng, Du et al. 2014 | China | Cross-section | Abdominal ultrasonography | NA | 1779 subjects | NAFLD was associated with hypertension: OR=1.49 (95% CI: 1.10-2.03); Lean-NAFLD was more strongly associated with hypertension: OR=1.72 (95% CI: 1-2.96) |
| Huh et al. 2015 | Korea | Prospective Cohort | Fatty liver index | 2.6 years | 1521 subjects without hypertension | Incident hypertension increased in a graded manner with NAFLD, 30–59 year old: OR: 1.83 (95% CI: 1.16~2.88); >60 year old: OR=2.09 (95% CI: 1.08~4.055) |
| Long et al. 2016 | USA | Cross-section | Abdominal ultrasonography | NA | 575 subjects with NAFLD | Participants with a high risk for advanced fibrosis by the NFS an increased odds of hypertension (OR=2.92, 95% CI: 1.35–6.34) compared to those with low risk of fibrosis. |
| Zhou et al 2018 | China | Prospective Cohort | Fatty liver index | 9 years | 4686 subjects without hypertension | NAFLD was associated with increased risk of incident hypertension: HR=1.51 (95% CI: 1.27–1.80) |
| Brunner et al. 2019 | USA | Prospective Cohort | Computed tomography | 6.2 years | 654 subjects with or without NAFLD | Increase in liver fat was associated with hypertension: OR=1.22 (95% CI: 0.96–1.55) |
| Roh et al. 2020 | Korea | Prospective Cohort | Fatty liver index | 5.2 years | 334280 healthy participants | High FLI score was associated with incident hypertension: HR=2.33 (95% CI: 2.218–2.448) |
| Ma et al. 2017 | USA | Prospective cohort | Abdominal scan with MDCT | 6 years | 1015 subjects with and without prevalent fatty liver | Hypertension was an independent predictor of incident NAFLD: OR=3.34 (95% CI: 2.04-5.49); NAFLD was associated with increased risks of incident hypertension: OR=1.42 (95% CI: 1.15-1.76) |
| Liu et al. 2018 | China | Prospective Cohort | Abdominal ultrasonography | 5 years | 6704 subjects without hypertension and 9328 subjects without NAFLD | Development and persistence of NAFLD was substantially associated with an increased: OR=1.49 (95% CI: 1.26–1.76); Incident hypertension was associated with risk of incident NAFLDD in subjects without diabetes or obesity: OR=1.45 (95% CI: 1.23–1.71) |

Supplementary Table 2. Obtained related genes to hypertension and NAFLD.

| Hypertension | NAFLD | Fibrosis | Inflammation |
| --- | --- | --- | --- |
| TNF | SREBF1 | TGFB1 | TNF |
| IL6 | CPT1A | TNF | IL1B |
| IL1B | PPARA | COL1A1 | IL6 |
| FOS | TGFB1 | AKT1 | PTGS2 |
| CAT | NFE2L2 | PTGS2 | NOS2 |
| REN | CAT | AGT | HMOX1 |
| BCL2 | ADIPOQ | SOD2 | CCL2 |
| NOS2 | LEP | IL6 | CXCL8 |
| TGFB1 | SCARB1 | ACTA2 | IL10 |
| RELA | LDLR | COL3A1 | ICAM1 |
| EDN1 | CYP2E1 | STAT3 | AKT1 |
| SOD2 | PDK4 | COL1A2 | MPO |
| JUN | NQO1 | CTNNB1 | VEGFA |
| CCL2 | CD14 | TLR4 | MMP9 |
| TP53 | ABCC2 | ALB | SOD1 |
| MMP2 | NR1H4 | IL4 | MMP2 |
| MMP9 | TRIB3 | VEGFA | CXCL2 |
| GSK3B | AHR | MMP2 | PARP1 |
| NR3C1 | VLDLR | THBS1 | IL1A |
| COL1A1 | FAS | CCL2 | TIMP1 |
| TH | XBP1 | SIRT1 | CCL3 |
| SERPINE1 | CYP1A2 | DCN | TLR4 |
| ICAM1 | PRKCA | CYP1A2 | EDN1 |
| LEP | IL1A | SLC27A2 | EGR1 |
| VCAM1 | ALDH1A1 | ADRB2 | CSF2 |
| FN1 | GSTP1 | LEPR | CASP1 |
| ADIPOQ | ALDH2 | CD14 | AGT |
| ACE | IL4 | PDGFB | IKBKB |
| GPX1 | PTEN | AGTR1A | LCN2 |
| TIMP1 | GSTA1 | ATM | AHR |
| GCLC | SERPINB2 | REN | CRP |
| NCF1 | PNPLA3 | LGALS3 | TFRC |
| CYBB | CSF2 | CCN2 | TLR2 |
| CYP1A1 | STC2 | GPX4 | IL13 |
| GSTP1 | NR5A2 | VIM | CCL4 |
| LHB | GSTM1 | ABCB4 | CCL11 |
| CCN2 | PEMT | ANGPT1 | IL17A |
| CYBA | ACE | CYP19A1 | NGF |
| COL3A1 | AHCY | TIMP1 | MYD88 |
| LPL | ALDH4A1 | CYBA | TAC1 |
| NOX4 | PRKACA | MIR21 | HMGB1 |
| CD36 | ABCB4 | SPP1 | TNFAIP3 |
| DRD2 | GSTT1 | SMAD3 | NLRP3 |
| CRP | LIF | PTEN | EIF4EBP1 |
| TLR4 | CYP17A1 | IGFBP1 | PLAUR |
| APOE | GNMT | GPT | MIF |
| CAV1 | PRKCD | IGFBP2 | PTGES |
| GJA1 | MMP1 | SFRP1 | TF |
| ADRB1 | JAK2 | SPARC | AGER |
| CXCL2 | F2 | AHR | MBP |
| AR | IKBKG | FLT1 | ZFP36 |
| ABCC1 | PRKCE | TAC1 | F2R |
| UCP2 | ALDH1B1 | F3 | NR1H4 |
| EDNRA | FOLR2 | NPPA | PROCR |
| PRKCD | TNFRSF1B | RELA | CALCA |
| PTEN | KLB | F2 | IRF7 |
| AVP | TM6SF2 | CP | COL2A1 |
| HSD11B2 | ADH4 | ATP6AP2 | GATA3 |
| PLAT | ATP5B | CYP27A1 | ABCB4 |
| ANXA1 | LAMA1 | F2R | GAL |
| AHR | B3GAT1 | FECH | KYNU |
| KNG1 | EIF2AK1 | LGALS1 | PTGER4 |
| AGTR2 | PRF1 | SREBF1 | TRPV1 |
| MTOR | RDX | TIMP2 | ANGPT1 |
| AGTR1 | IL3 | NFKBIA | BDKRB1 |
| EPO | ADH1B | ALAD | CYP3A23/3A1 |
| APOA1 | ADH1A | SERPINH1 | MIR21 |
| HSD11B1 | RAG2 | IL1B | MVK |
| RPS6KB1 | PPARD | CD9 | BDKRB2 |
| VDR | SIRT1 | MMP9 | RORC |
| GAL | FGF21 | TGFBR1 | F2RL1 |
| IER3 |  | PIK3CG | SLC22A5 |
| ATP1A1 |  | C1QB | CYP2B1 |
| ALAD |  | ABCC2 | SCGB1A1 |
| GSTT1 |  | LOX | ATP7B |
| LEPR |  | TNFRSF1B | CYP3A2 |
| IKBKB |  | FAS | MIR34A |
| GCH1 |  | APP | PLAA |
| TPM1 |  | NOS3 | TBX21 |
| GNAS |  | FBN1 | CXCR3 |
| DUSP5 |  | IGFBP3 | EFNB1 |
| ADRA2A |  | LGALS3BP | WDR1 |
| VWF |  | NPR1 | IDO1 |
| CBS |  | SLC10A1 | CD3E |
| AQP1 |  | DHCR7 | ASIC1 |
| SLC12A2 |  | CXCL10 | IL17RA |
| HP |  | CD36 | CYP2B2 |
| ECE1 |  | LUM | TP73 |
| PLG |  | COL5A2 | SULT2B1 |
| SCNN1A |  | LEP | SELENOS |
| GNRH1 |  | PDGFD | CASP3 |
| MYD88 |  | BIRC3 | ASIC2 |
| GCG |  | PDGFRB | TSC2 |
| SELP |  | LOXL1 | TREX1 |
| PTH |  | CDKN1A | MIR22 |
| OLR1 |  | EGF | OVAL |
| TGM2 |  | A2M | IFNG |
| CACNA1C | | SERPINA6 | TLR9 |
| BDKRB2 |  | CCL5 | TRPA1 |
| NEDD4L |  | CYP2C11 | TNFSF15 |
| ATP1A2 |  | ARNT | TGFB1 |
| ALOX15 |  | MAP2K7 | MAPK3 |
| CYP4A10 |  | CD68 | MAPK1 |
| SLC6A2 |  | CXCL1 | MASP2 |
| PPBP |  | NR0B2 | CLN6 |
| AGT |  | COL4A1 | ASIC3 |
| SLC12A3 |  | SLC22A8 | TOP2A |
| MDH1 |  | GC | NOS3 |
| NPR1 |  | LBP | LBP |
| EDN3 |  | CCNA2 | ALOX5 |
| AOC1 |  | HSD11B2 | SOD2 |
| PROC |  | PEMT | LTB4R |
| TRH |  | S100A9 | TGFBI |
| GUCY1A1 | | HMGCR | ADM |
| SLC9A3R2 | | MYC | CXCR2 |
| ADCY5 |  | GPAM | MT1 |
| ELN |  | PLOD2 | MIR217 |
| ATP2B1 |  | GOT1 | APP |
| SCNN1G |  | ITGB1 | MT2 |
| GSTA5 |  | CYBB | ADORA2A |
| GSTT2 |  | PDGFA | TACR1 |
| SCNN1B |  | LPL | ATP5F1A |
| CACNA2D1 | | SCD2 | HSPD1 |
| AOC3 |  | RGN | LTA |
| GJA4 |  | AQP7 | RPL4 |
| NPPA |  | SLC20A1 | ADORA2B |
| ITM2B |  | TRIB3 | ABCB1B |
| WNK1 |  | ABCB1 | OTC |
| F12 |  | F5 | HSPA9 |
| FXYD2 |  | TGFB3 | CPS1 |
| TACR1 |  | MKI67 | IFNL3 |
| HTR2B |  | PTGS1 | CIRBP |
| ADD1 |  | TAGLN2 | REN2 |
| CXCR2 |  | ABCG5 | RPL9 |
| GRK2 |  | FGF7 | GJB2 |
| AGTR1B |  | KRT19 | IFNA2 |
| ORAI1 |  | THG1L | HLA-DRA |
| GLP1R |  | JUND | ANG |
| GJA5 |  | PCK1 | AQP1 |
| NPTN |  | LDHB | C5AR1 |
| ERAP1 |  | CCNB2 | CISH |
| VAV3 |  | ADD3 | GC |
| STK11 |  | CDK1 | NLRC4 |
| REG3G |  | ABCC5 | NLRP1 |
| TRPC5 |  | ANXA3 | OTULIN |
| F11 |  | ACAT1 | PLCG2 |
| STIM1 |  | ATP7B | PSMB8 |
| KCNJ1 |  | FOXA2 | TIRAP |
| UMOD |  | FSTL1 | PPARG |
| GNB3 |  | LAMC2 | STAT3 |
| RALBP1 |  | G0S2 | LEP |
| WNK4 |  | CASP1 | PPARA |
| SLC6A18 |  | ATF3 | BDNF |
| MC2R |  | AP1S2 | FGF2 |
| INPPL1 |  | CAV1 | IL1RN |
| CRHR2 |  | CHSY1 | TNFRSF11B |
| HRH3 |  | PLAT | ADIPOQ |
| TRPC3 |  | PPP1R3C | APOA1 |
| CELA2A |  | AFP | NPPB |
| ATP2B3 |  | NOX4 | TGFA |
| CMA1 |  | GHR | JAK2 |
| MCPT1 |  | CYP3A11 | DUSP10 |
| NOS3 |  | CTSS | LTF |
| KCNMB1 |  | PLAU | TSLP |
| CETP |  | FOS | EPO |
| REN2 |  | THBD | PTPN1 |
| GP1BA |  | RDH10 | STS |
| UTS2 |  | PDGFRA | CHRNA4 |
| CLCNKA |  | PLSCR1 | SH2B3 |
| CYP3A5 |  | SULT1E1 | SERPINA3K |
| MOXD2 |  | LCN2 | CCK |
| CYP17A1 |  | CDO1 | CHRNB2 |
| MYH9 |  | COTL1 | CRHR2 |
| ATP1B1 |  | CASP12 | GALNS |
| CALY |  | ITGB2 | UCN |
| NR3C2 |  | CXCL16 | OXT |
| PDE3A |  | NOCT | POMC |
| PTGIS |  | PLTP | UCN3 |
| RGS5 |  | VASP | CSF3 |
| YY1AP1 |  | G6PC | PROC |
| PTGS2 |  | ALDH1A1 | AMN |
| HMOX1 |  | GBP2 | MCAM |
| SOD1 |  | UGCG | PNOC |
| PPARG |  | BAMBI | PLAT |
| POMC |  | SLC25A4 |  |
| INS |  | ISYNA1 |  |
| NPPB |  | KRT18 |  |
| IGF1 |  | VTN |  |
| PPARA |  | LY96 |  |
| SOD3 |  | ADA |  |
| ACE2 |  | GJA1 |  |
| ALB |  | PLA2G4A |  |
| HIF1A |  | TNFRSF12A | |
| CALCA |  | NUPR1 |  |
| RGS2 |  | ADH4 |  |
| OXT |  | ASNS |  |
| LTF |  | S100A6 |  |
| STK39 |  | SMAD2 |  |
| KLK1 |  | GSTA2 |  |
| PTPN1 |  | OLFML3 |  |
| ATOX1 |  | ENC1 |  |
| GJC1 |  | ACSL1 |  |
| UCN |  | TYROBP |  |
| OXSR1 |  | UBD |  |
|  |  | LGMN |  |
|  |  | PTPRO |  |
|  |  | GCK |  |
|  |  | CXCL12 |  |
|  |  | PROCR |  |
|  |  | CD163 |  |
|  |  | PLIN2 |  |
|  |  | ENPP2 |  |
|  |  | SULT2A1 |  |
|  |  | FAT1 |  |
|  |  | GRN |  |
|  |  | COL6A1 |  |
|  |  | GSN |  |
|  |  | MIR29C |  |
|  |  | HPSE |  |
|  |  | ANXA2 |  |
|  |  | SMOC1 |  |
|  |  | SOX4 |  |
|  |  | ADHFE1 |  |
|  |  | ADGRE1 |  |
|  |  | CSF1R |  |
|  |  | COLEC12 |  |
|  |  | MGP |  |
|  |  | RGS2 |  |
|  |  | ADRA2C |  |
|  |  | NREP |  |
|  |  | FGF2 |  |
|  |  | DCXR |  |
|  |  | PCOLCE |  |
|  |  | NID2 |  |
|  |  | PRNP |  |
|  |  | CDKN3 |  |
|  |  | CASP7 |  |
|  |  | NT5E |  |
|  |  | SLC17A2 |  |
|  |  | TMSB10 |  |
|  |  | ABCB1A |  |
|  |  | CEBPD |  |
|  |  | FGFR2 |  |
|  |  | S100A11 |  |
|  |  | SERPINA1 | |
|  |  | ERN1 |  |
|  |  | IFITM1 |  |
|  |  | FCER1G |  |
|  |  | RAMP1 |  |
|  |  | APAF1 |  |
|  |  | KLF6 |  |
|  |  | TPM3 |  |
|  |  | PFN1 |  |
|  |  | LTBP1 |  |
|  |  | PRKCB |  |
|  |  | SOCS2 |  |
|  |  | KRT8 |  |
|  |  | STAU2 |  |
|  |  | ABCG8 |  |
|  |  | MAP3K8 |  |
|  |  | CAMK2D |  |
|  |  | TNFAIP8 |  |
|  |  | CYP2R1 |  |
|  |  | NUDT16 |  |
|  |  | IL18 |  |
|  |  | SELENOP |  |
|  |  | FMNL2 |  |
|  |  | CAR3 |  |
|  |  | C1QA |  |
|  |  | FABP7 |  |
|  |  | SHCBP1 |  |
|  |  | APOA4 |  |
|  |  | IKBKG |  |
|  |  | CEMIP2 |  |
|  |  | HDAC2 |  |
|  |  | ANXA1 |  |
|  |  | MSN |  |
|  |  | CCNG1 |  |
|  |  | ILK |  |
|  |  | SDS |  |
|  |  | CRYAB |  |
|  |  | ARPC1B |  |
|  |  | PRKCD |  |
|  |  | EZR |  |
|  |  | ADM |  |
|  |  | JAK2 |  |
|  |  | OBP3 |  |
|  |  | ISG20 |  |
|  |  | DHRS3 |  |
|  |  | COL4A2 |  |
|  |  | TUBB6 |  |
|  |  | TRIM24 |  |
|  |  | CNR2 |  |
|  |  | NDRG1 |  |
|  |  | AADAT |  |
|  |  | SMOC2 |  |
|  |  | SH3BGRL3 | |
|  |  | AKR1B1 |  |
|  |  | NFKBIZ |  |
|  |  | TAX1BP3 |  |
|  |  | CASP4 |  |
|  |  | GJA4 |  |
|  |  | ALDH2 |  |
|  |  | TPM1 |  |
|  |  | INHBC |  |
|  |  | GK |  |
|  |  | SPTAN1 |  |
|  |  | SLC30A10 | |
|  |  | DDC |  |
|  |  | HOPX |  |
|  |  | SERPING1 | |
|  |  | ACOT9 |  |
|  |  | REEP5 |  |
|  |  | TAP2 |  |
|  |  | TSC22D1 |  |
|  |  | CPS1 |  |
|  |  | TUSC3 |  |
|  |  | PSMB9 |  |
|  |  | GZMA |  |
|  |  | TES |  |
|  |  | ZEB2 |  |
|  |  | CD83 |  |
|  |  | EMP1 |  |
|  |  | PDZRN3 |  |
|  |  | IGF2BP3 |  |
|  |  | MYO5A |  |
|  |  | NID1 |  |
|  |  | RBM3 |  |
|  |  | TPM4 |  |
|  |  | ADAM17 |  |
|  |  | RTN4 |  |
|  |  | IQGAP1 |  |
|  |  | FLI1 |  |
|  |  | PKLR |  |
|  |  | TFPI2 |  |
|  |  | RAB31 |  |
|  |  | IGF2R |  |
|  |  | PFKP |  |
|  |  | BMF |  |
|  |  | MT2A |  |
|  |  | SERPINB9 | |
|  |  | ANGPTL3 |  |
|  |  | ID4 |  |
|  |  | KCNJ8 |  |
|  |  | NDRG2 |  |
|  |  | ITGB3 |  |
|  |  | GSTM1 |  |
|  |  | GSTA3 |  |
|  |  | WIPF1 |  |
|  |  | RNASE4 |  |
|  |  | ACKR3 |  |
|  |  | ANXA5 |  |
|  |  | ECT2 |  |
|  |  | PTPRD |  |
|  |  | CCL6 |  |
|  |  | INSIG2 |  |
|  |  | MGMT |  |
|  |  | BBOX1 |  |
|  |  | FKBP1A |  |
|  |  | ARL8A |  |
|  |  | CMTM3 |  |
|  |  | PRLR |  |
|  |  | THYN1 |  |
|  |  | DRAM1 |  |
|  |  | CD86 |  |
|  |  | SMC2 |  |
|  |  | FHL2 |  |
|  |  | GPNMB |  |
|  |  | MT1A |  |
|  |  | SLC19A2 |  |
|  |  | LAPTM5 |  |
|  |  | NAT8 |  |
|  |  | FMO1 |  |
|  |  | PPP1R18 |  |
|  |  | MID1IP1 |  |
|  |  | HJV |  |
|  |  | CD38 |  |
|  |  | SLC39A8 |  |
|  |  | IL33 |  |
|  |  | FXYD5 |  |
|  |  | RABGAP1L | |
|  |  | ABCG1 |  |
|  |  | COL12A1 |  |
|  |  | LIMK2 |  |
|  |  | ZFYVE16 |  |
|  |  | PSTPIP1 |  |
|  |  | LBH |  |
|  |  | FGF15 |  |
|  |  | SLC4A4 |  |
|  |  | PTPN18 |  |
|  |  | ARRB1 |  |
|  |  | AMACR |  |
|  |  | NOS2 |  |
|  |  | AHNAK |  |
|  |  | VWF |  |
|  |  | SRD5A1 |  |
|  |  | NQO2 |  |
|  |  | PPP1R3B |  |
|  |  | LIN7A |  |
|  |  | MME |  |
|  |  | ABLIM3 |  |
|  |  | GCAT |  |
|  |  | CYP3A13 |  |
|  |  | C8G |  |
|  |  | NPDC1 |  |
|  |  | IL7R |  |
|  |  | FGR |  |
|  |  | CAPG |  |
|  |  | ACOX2 |  |
|  |  | LRRFIP1 |  |
|  |  | GCNT1 |  |
|  |  | BDH1 |  |
|  |  | RARRES1 |  |
|  |  | KYNU |  |
|  |  | BICD2 |  |
|  |  | SKAP2 |  |
|  |  | SLC25A30 | |
|  |  | PLPP2 |  |
|  |  | INMT |  |
|  |  | ADH1 |  |
|  |  | GFRA1 |  |
|  |  | IFI35 |  |
|  |  | NRG1 |  |
|  |  | KMO |  |
|  |  | APPL1 |  |
|  |  | DBNDD2 |  |
|  |  | SERPINA3K | |
|  |  | ABAT |  |
|  |  | RHOQ |  |
|  |  | LRAT |  |
|  |  | OTUB2 |  |
|  |  | TMBIM1 |  |
|  |  | MIR503 |  |
|  |  | SLC13A4 |  |
|  |  | CD74 |  |
|  |  | SPSB4 |  |
|  |  | CYP2A2 |  |
|  |  | IGFALS |  |
|  |  | ANXA4 |  |
|  |  | EPB41L2 |  |
|  |  | WLS |  |
|  |  | ADRA1A |  |
|  |  | NPC1 |  |
|  |  | CXCL9 |  |
|  |  | IFRD1 |  |
|  |  | LTB |  |
|  |  | FAM43A |  |
|  |  | CES2 |  |
|  |  | FHL1 |  |
|  |  | ALDH1A7 |  |
|  |  | CD63 |  |
|  |  | IL2RG |  |
|  |  | PALMD |  |
|  |  | HPCAL1 |  |
|  |  | SOAT1 |  |
|  |  | MTMR7 |  |
|  |  | CYP26A1 |  |
|  |  | AKR1C3 |  |
|  |  | ABCA8A |  |
|  |  | ACHE |  |
|  |  | ACP5 |  |
|  |  | B4GALT5 |  |
|  |  | NFE2L2 |  |
|  |  | BGN |  |
|  |  | IL18BP |  |
|  |  | RELL1 |  |
|  |  | CDC42SE1 | |
|  |  | GNAI2 |  |
|  |  | NMI |  |
|  |  | KANK1 |  |
|  |  | RCN1 |  |
|  |  | CHRNA1 |  |
|  |  | EPCAM |  |
|  |  | FABP2 |  |
|  |  | HAO2 |  |
|  |  | OAT |  |
|  |  | DUSP6 |  |
|  |  | TMEM165 | |
|  |  | CD53 |  |
|  |  | PLEKHH1 |  |
|  |  | C1QC |  |
|  |  | INHBE |  |
|  |  | RGS5 |  |
|  |  | YPEL3 |  |
|  |  | PRODH2 |  |
|  |  | CSAD |  |
|  |  | HCK |  |
|  |  | WFDC2 |  |
|  |  | PTPN1 |  |
|  |  | CYP2C |  |
|  |  | FAM89A |  |
|  |  | GLT8D1 |  |
|  |  | MYO1C |  |
|  |  | OTC |  |
|  |  | GET1 |  |
|  |  | CROT |  |
|  |  | IFNGR1 |  |
|  |  | PMEPA1 |  |
|  |  | COL5A1 |  |
|  |  | CPQ |  |
|  |  | PLA2G12A | |
|  |  | AIDA |  |
|  |  | CNR1 |  |
|  |  | GGH |  |
|  |  | TCF4 |  |
|  |  | DIO1 |  |
|  |  | NEK6 |  |
|  |  | TSPAN8 |  |
|  |  | RCN2 |  |
|  |  | RGS10 |  |
|  |  | SRI |  |
|  |  | PSMB8 |  |
|  |  | ADRA1B |  |
|  |  | CTLA2A |  |
|  |  | HSD17B2 |  |
|  |  | COL6A2 |  |
|  |  | APBB1IP |  |
|  |  | PLEK |  |
|  |  | EMB |  |
|  |  | PLSCR2 |  |
|  |  | RTP3 |  |
|  |  | TBC1D9 |  |
|  |  | GLS |  |
|  |  | RBL2 |  |
|  |  | CYP17A1 |  |
|  |  | GLIPR1 |  |
|  |  | RASSF4 |  |
|  |  | CTSE |  |
|  |  | CCDC126 |  |
|  |  | DHTKD1 |  |
|  |  | METRNL |  |
|  |  | AADAC |  |
|  |  | SLC25A13 | |
|  |  | VAV1 |  |
|  |  | VSIR |  |
|  |  | OSGIN1 |  |
|  |  | PTER |  |
|  |  | CXADR |  |
|  |  | CLEC4A3 |  |
|  |  | UGT2B |  |
|  |  | SERPINA4 | |
|  |  | MEOX2 |  |
|  |  | PRUNE2 |  |
|  |  | VIPR1 |  |
|  |  | AEN |  |
|  |  | FGL2 |  |
|  |  | GLS2 |  |
|  |  | NFKBIE |  |
|  |  | PLAC8 |  |
|  |  | DOCK10 |  |
|  |  | PAQR9 |  |
|  |  | CFD |  |
|  |  | IGSF6 |  |
|  |  | RAC2 |  |
|  |  | SERPINA7 | |
|  |  | FYN |  |
|  |  | STEAP4 |  |
|  |  | RAB8B |  |
|  |  | DHRS7 |  |
|  |  | EXTL1 |  |
|  |  | LPXN |  |
|  |  | PYCR1 |  |
|  |  | ACTN1 |  |
|  |  | MAP1LC3A | |
|  |  | RAB30 |  |
|  |  | GULO |  |
|  |  | CD5L |  |
|  |  | GBP4 |  |
|  |  | HEXIM1 |  |
|  |  | YWHAG |  |
|  |  | AKR1C1 |  |
|  |  | RECK |  |
|  |  | SMAD4 |  |
|  |  | FAHD1 |  |
|  |  | SRR |  |
|  |  | ZFP37 |  |
|  |  | HAL |  |
|  |  | S1PR1 |  |
|  |  | ARHGDIB |  |
|  |  | STK10 |  |
|  |  | CD48 |  |
|  |  | SRGN |  |
|  |  | CIDEA |  |
|  |  | MALAT1 |  |
|  |  | GNPDA1 |  |
|  |  | ARRB2 |  |
|  |  | SLC37A4 |  |
|  |  | FLNA |  |
|  |  | ALDH1L2 |  |
|  |  | CKLF |  |
|  |  | SELENOM | |
|  |  | APOBEC1 | |
|  |  | IFI204 |  |
|  |  | HSD17B6 |  |
|  |  | AGL |  |
|  |  | CELA1 |  |
|  |  | ST14 |  |
|  |  | TMEM43 |  |
|  |  | CES3 |  |
|  |  | GGTA1 |  |
|  |  | NCKAP1L |  |
|  |  | LOXL2 |  |
|  |  | PCK2 |  |
|  |  | MDH1 |  |
|  |  | FBXO9 |  |
|  |  | MVP |  |
|  |  | RAB32 |  |
|  |  | ADORA2A | |
|  |  | TMEM65 |  |
|  |  | HELLS |  |
|  |  | HPGDS |  |
|  |  | SLC23A1 |  |
|  |  | TM6SF1 |  |
|  |  | APON |  |
|  |  | SLC46A1 |  |
|  |  | CHRM3 |  |
|  |  | IFI27L2B |  |
|  |  | NFKB1 |  |
|  |  | FTCD |  |
|  |  | JDP2 |  |
|  |  | S100A10 |  |
|  |  | PTGER2 |  |
|  |  | INPP5D |  |
|  |  | IVD |  |
|  |  | UST5R |  |
|  |  | CLP1 |  |
|  |  | GNGT2 |  |
|  |  | SOCS1 |  |
|  |  | OLFML2B | |
|  |  | PRODH |  |
|  |  | ASAH1 |  |
|  |  | DPYS |  |
|  |  | PXDN |  |
|  |  | STARD13 |  |
|  |  | ZDHHC2 |  |
|  |  | AFM |  |
|  |  | MFAP4 |  |
|  |  | SLC25A24 | |
|  |  | ZDHHC13 | |
|  |  | GNA14 |  |
|  |  | SGCB |  |
|  |  | SLAMF9 |  |
|  |  | CYP3A9 |  |
|  |  | SLC43A2 |  |
|  |  | PSMB10 |  |
|  |  | RSRP1 |  |
|  |  | ALDH1L1 |  |
|  |  | APMAP |  |
|  |  | ELOVL5 |  |
|  |  | SAMD11 |  |
|  |  | FZD8 |  |
|  |  | DUSP16 |  |
|  |  | AGFG1 |  |
|  |  | FAM102B |  |
|  |  | MOB3A |  |
|  |  | HIF1A |  |
|  |  | HAO1 |  |
|  |  | LGALSL |  |
|  |  | RGS3 |  |
|  |  | TMEM67 |  |
|  |  | ACBD4 |  |
|  |  | AIF1 |  |
|  |  | BCR |  |
|  |  | XPNPEP2 |  |
|  |  | AP3S1 |  |
|  |  | COQ10A |  |
|  |  | MIS12 |  |
|  |  | RAB40B |  |
|  |  | ITGB6 |  |
|  |  | CLIC1 |  |
|  |  | FXYD6 |  |
|  |  | PTPRC |  |
|  |  | LRRC28 |  |
|  |  | STAC3 |  |
|  |  | MYL9 |  |
|  |  | CD24 |  |
|  |  | CRY1 |  |
|  |  | PLEKHB1 |  |
|  |  | IL2RB |  |
|  |  | IRF8 |  |
|  |  | HAGH |  |
|  |  | PAMR1 |  |
|  |  | DAO |  |
|  |  | MMP15 |  |
|  |  | MAGEE1 |  |
|  |  | CELF2 |  |
|  |  | NPAS2 |  |
|  |  | PC |  |
|  |  | CTSZ |  |
|  |  | CAR5A |  |
|  |  | NAP1L5 |  |
|  |  | CYP2D4 |  |
|  |  | RT1-DA |  |
|  |  | GASK1B |  |
|  |  | MYADM |  |
|  |  | TKFC |  |
|  |  | ASPN |  |
|  |  | ENPEP |  |
|  |  | HOXA3 |  |
|  |  | UNC5CL |  |
|  |  | BLVRA |  |
|  |  | ALDH1B1 |  |
|  |  | IGFBP7 |  |
|  |  | ARMCX1 |  |
|  |  | PRTG |  |
|  |  | RMDN2 |  |
|  |  | SLC39A6 |  |
|  |  | PYCARD |  |
|  |  | CTTNBP2NL | |
|  |  | HYKK |  |
|  |  | MYO1F |  |
|  |  | GULP1 |  |
|  |  | SELENOW | |
|  |  | UNC93B1 |  |
|  |  | APOL9A |  |
|  |  | TMEM184B | |
|  |  | AKTIP |  |
|  |  | PTRH1 |  |
|  |  | ATP6V1D |  |
|  |  | MGAT5 |  |
|  |  | TRPM7 |  |
|  |  | CD7 |  |
|  |  | CLEC4A |  |
|  |  | CNPY4 |  |
|  |  | L3HYPDH |  |
|  |  | ANKS6 |  |
|  |  | YWHAZ |  |
|  |  | MINDY3 |  |
|  |  | SERPINA3M | |
|  |  | CD276 |  |
|  |  | C5AR1 |  |
|  |  | COMMD1 | |
|  |  | ATP10D |  |
|  |  | INPPL1 |  |
|  |  | GPX8 |  |
|  |  | NECTIN2 |  |
|  |  | SEZ6 |  |
|  |  | CRTAP |  |
|  |  | TTC36 |  |
|  |  | FCGR3A |  |
|  |  | LCP2 |  |
|  |  | PTAFR |  |
|  |  | CFP |  |
|  |  | FXYD2 |  |
|  |  | ABCG2 |  |
|  |  | RDH13 |  |
|  |  | SLFN2 |  |
|  |  | PQLC3 |  |
|  |  | TRIM59 |  |
|  |  | CXCL13 |  |
|  |  | NAGK |  |
|  |  | LGALS2 |  |
|  |  | SLC7A1 |  |
|  |  | ADORA3 |  |
|  |  | DPP7 |  |
|  |  | SEMA6D |  |
|  |  | SLC15A3 |  |
|  |  | TMEM106A | |
|  |  | VOPP1 |  |
|  |  | LY86 |  |
|  |  | PTN |  |
|  |  | PKD2 |  |
|  |  | TIAL1 |  |
|  |  | TRAFD1 |  |
|  |  | RPH3AL |  |
|  |  | RGD1305347 | |
|  |  | RGD1307603 | |
|  |  | AXL |  |
|  |  | DYSF |  |
|  |  | ARHGAP30 | |
|  |  | ATRN |  |
|  |  | PSME2 |  |
|  |  | TRAK2 |  |
|  |  | TNFRSF1A | |
|  |  | TSPO |  |
|  |  | FOLR2 |  |
|  |  | FRY |  |
|  |  | KRT7 |  |
|  |  | DEPDC7 |  |
|  |  | OTULINL |  |
|  |  | AKR1C19 |  |
|  |  | CYSTM1 |  |
|  |  | SLA |  |
|  |  | ITGA2B |  |
|  |  | ANO6 |  |
|  |  | CNP |  |
|  |  | LCP1 |  |
|  |  | GCHFR |  |
|  |  | TRPV1 |  |
|  |  | HIP1 |  |
|  |  | R3HDM2 |  |
|  |  | ICAM2 |  |
|  |  | RT1-CE5 |  |
|  |  | SNAP23 |  |
|  |  | ITGAL |  |
|  |  | PLIN3 |  |
|  |  | KEAP1 |  |
|  |  | NIPAL2 |  |
|  |  | SLC17A1 |  |
|  |  | ZBTB4 |  |
|  |  | RGD1564865 | |
|  |  | SPIN2A |  |
|  |  | PPAP2A |  |
|  |  | TFEC |  |
|  |  | BLOC1S2 |  |
|  |  | RNF39 |  |
|  |  | SECTM1B |  |
|  |  | WFDC21 |  |
|  |  | CERK |  |
|  |  | SNAP29 |  |
|  |  | DIPK2A |  |
|  |  | GPR68 |  |
|  |  | NLRC5 |  |
|  |  | SNX7 |  |
|  |  | BCL2A1D |  |
|  |  | RIT1 |  |
|  |  | SLC51A |  |
|  |  | CA3 |  |
|  |  | CYP2T1 |  |
|  |  | SHISA3 |  |
|  |  | B9D1 |  |
|  |  | SMC5 |  |
|  |  | CD200R1 |  |
|  |  | PRR13 |  |
|  |  | CLEC4F |  |
|  |  | GALC |  |
|  |  | SDR42E1 |  |
|  |  | SYNRG |  |
|  |  | SGK2 |  |
|  |  | PEA15A |  |
|  |  | OLR59 |  |
|  |  | LTA4H |  |
|  |  | ARHGAP9 | |
|  |  | RT1-DB1 |  |
|  |  | SELENBP2 | |
|  |  | RT1-DMA | |
|  |  | PLD4 |  |
|  |  | SLC51B |  |
|  |  | LURAP1L |  |
|  |  | DOCK7 |  |
|  |  | TMEM178 | |
|  |  | ROBO2 |  |
|  |  | RGD1562339 | |
|  |  | SLC22A23 | |
|  |  | CNDP1 |  |
|  |  | IYD |  |
|  |  | NSMF |  |
|  |  | KTN1 |  |
|  |  | RT1-BA |  |
|  |  | PRTFDC1 |  |
|  |  | RGD1566227 | |
|  |  | ARL11 |  |
|  |  | GMFG |  |
|  |  | RNASEL |  |
|  |  | SIGLEC5 |  |
|  |  | TOR4A |  |
|  |  | MIR885 |  |
|  |  | MPO |  |
|  |  | NCOR2 |  |
|  |  | SLC44A2 |  |
|  |  | TRF |  |
|  |  | TCF20 |  |
|  |  | MIR217 |  |
|  |  | EIF4E3 |  |
|  |  | FCGR1A |  |
|  |  | RDH2 |  |
|  |  | NBEA |  |
|  |  | CDH5 |  |
|  |  | MRO |  |
|  |  | RGD1309534 | |
|  |  | LMO2 |  |
|  |  | IL12B |  |
|  |  | MIR376C |  |
|  |  | FCN1 |  |
|  |  | RT1-M3-1 | |
|  |  | PRAF2 |  |
|  |  | RCSD1 |  |
|  |  | LHFPL6 |  |
|  |  | RT1-DMB | |
|  |  | SASH3 |  |
|  |  | CDKN2AIPNL | |
|  |  | MYOC |  |
|  |  | ERAP1 |  |
|  |  | IL12A |  |
|  |  | C7 |  |
|  |  | NRROS |  |
|  |  | RGD1561149 | |
|  |  | CLEC4A1 |  |
|  |  | FAM167B |  |
|  |  | CD200R2 |  |
|  |  | SMARCC1 | |
|  |  | CSGALNACT1 | |
|  |  | MAPK14 |  |
|  |  | RMND5B |  |
|  |  | TYK2 |  |
|  |  | MIR30C2 |  |
|  |  | ENTPD2 |  |
|  |  | SP140 |  |
|  |  | DUS1L |  |
|  |  | CRIP |  |
|  |  | CLIC2 |  |
|  |  | MIR30B |  |
|  |  | FMNL1 |  |
|  |  | U2SURP |  |
|  |  | MIR17 |  |
|  |  | CYP2E1 |  |
|  |  | ES2 |  |
|  |  | MBOAT7 |  |
|  |  | CAT |  |
|  |  | MIR141 |  |
|  |  | MIR764 |  |
|  |  | NSA2 |  |
|  |  | ICAM3 |  |
|  |  | MIR302C |  |
|  |  | RGD1563818 | |
|  |  | CXCR5 |  |
|  |  | SERPINF1 |  |
|  |  | SLC4A2 |  |
|  |  | RAN |  |
|  |  | HPCL2 |  |
|  |  | MIR215 |  |
|  |  | TSTD3 |  |
|  |  | S100A4 |  |
|  |  | STAT4 |  |
|  |  | CCL8 |  |
|  |  | TAGLN |  |
|  |  | PRDX2 |  |
|  |  | BCL2 |  |
|  |  | HMGN5B |  |
|  |  | PRDX1 |  |
|  |  | MIR3615 |  |
|  |  | PTRHD1 |  |
|  |  | MIR589 |  |
|  |  | MIR421 |  |
|  |  | MIR484 |  |
|  |  | MIR629 |  |
|  |  | RDH3 |  |
|  |  | MIR338 |  |
|  |  | MIR30C-1 | |
|  |  | MIR483 |  |
|  |  | MIR423 |  |
|  |  | MIRLET7F-2 | |
|  |  | MIR193B |  |
|  |  | MIR532 |  |
|  |  | CLEC16A |  |
|  |  | SERPINB5 | |
|  |  | NEDD4 |  |
|  |  | SPIB |  |
|  |  | MIR93 |  |
|  |  | ARHGDIA |  |
|  |  | TM6SF2 |  |
|  |  | EIF5A |  |
|  |  | CD3D |  |
|  |  | DENND1B | |
|  |  | MIR153 |  |
|  |  | SFN |  |
|  |  | FGG |  |
|  |  | MIF |  |
|  |  | S100A8 |  |
|  |  | FTH1 |  |
|  |  | PNPLA3 |  |
|  |  | CD80 |  |
|  |  | CYGB |  |
|  |  | RGD1309104 | |
|  |  | ACTRT2 |  |
|  |  | CRABP1 |  |
|  |  | FOXL2 |  |
|  |  | FTL |  |
|  |  | HBD |  |
|  |  | IFNL3 |  |
|  |  | KIF21A |  |
|  |  | LBR |  |
|  |  | MIR942 |  |
|  |  | MMEL1 |  |
|  |  | MYDGF |  |
|  |  | MYL6B |  |
|  |  | PHOX2A |  |
|  |  | POLG |  |
|  |  | TPSB2 |  |
|  |  | TUBB3 |  |
|  |  | HMOX1 |  |
|  |  | IL1RN |  |
|  |  | CCND2 |  |
|  |  | STAT1 |  |
|  |  | SPHK1 |  |
|  |  | HGF |  |
|  |  | CSF3 |  |
|  |  | IL10 |  |
|  |  | ADRA2A |  |
|  |  | IGF1 |  |
|  |  | IFNG |  |
|  |  | EDN1 |  |
|  |  | MMP13 |  |
|  |  | ADCY5 |  |
|  |  | TGFBR2 |  |
|  |  | ID2 |  |
|  |  | MMP1 |  |
|  |  | KLK1 |  |
|  |  | ADIPOQ |  |
|  |  | GH1 |  |
|  |  | IFNA1 |  |
|  |  | KNG1 |  |
|  |  | INS |  |
|  |  | MMP8 |  |
|  |  | MIR133A1 | |
|  |  | BMP7 |  |
|  |  | SENP2 |  |
|  |  | CXCL15 |  |
|  |  | POMC |  |
|  |  | PLG |  |
|  |  | YBX1 |  |
|  |  | OSM |  |
|  |  | IFNA2 |  |

Supplementary Table 3. GO and KEGG enrichment of overlapped genes between hypertension and NAFLD modules.

| Category | ID | Description | p.adjust | geneID |
| --- | --- | --- | --- | --- |
| biological process | GO:0043434 | response to peptide hormone | 3.95E-10 | 142/183/533/847/1244/1393/2950/3952/4780/5020/5465/5580/5728/7040/9370 |
| biological process | GO:0097305 | response to alcohol | 3.87E-07 | 142/196/847/1393/2950/3952/5020/5728/7040/9370 |
| biological process | GO:0071375 | cellular response to peptide hormone stimulus | 3.87E-07 | 142/183/533/1393/2950/3952/4780/5580/5728/7040/9370 |
| biological process | GO:0032868 | response to insulin | 8.46E-07 | 142/183/533/847/2950/3952/5465/5580/5728/9370 |
| biological process | GO:0032355 | response to estradiol | 8.46E-07 | 183/847/1393/2950/3952/5020/5728/7040 |
| biological process | GO:0014910 | regulation of smooth muscle cell migration | 8.46E-07 | 183/596/657/1636/2950/4780/9370 |
| biological process | GO:1901653 | cellular response to peptide | 1.12E-06 | 142/183/533/1393/2950/3952/4780/5580/5728/7040/9370 |
| biological process | GO:0014909 | smooth muscle cell migration | 1.12E-06 | 183/596/657/1636/2950/4780/9370 |
| biological process | GO:0014812 | muscle cell migration | 2.38E-06 | 183/596/657/1636/2950/4780/9370 |
| biological process | GO:0046677 | response to antibiotic | 2.38E-06 | 196/596/847/1393/2950/3952/4780/5580/5728/9370 |
| biological process | GO:0014823 | response to activity | 3.87E-06 | 183/847/3952/5020/5728/9370 |
| biological process | GO:0008217 | regulation of blood pressure | 4.36E-06 | 183/477/1636/1909/3952/5020/5465/9370 |
| biological process | GO:0010038 | response to metal ion | 5.05E-06 | 48/142/596/805/845/847/1244/1393/4780/5728 |
| biological process | GO:0007565 | female pregnancy | 5.67E-06 | 183/259/596/1244/1393/3952/5020/7040 |
| biological process | GO:0090322 | regulation of superoxide metabolic process | 5.94E-06 | 183/2950/4780/5580/7040 |
| biological process | GO:0032869 | cellular response to insulin stimulus | 1.24E-05 | 142/183/533/2950/3952/5580/5728/9370 |
| biological process | GO:0044706 | multi-multicellular organism process | 1.44E-05 | 183/259/596/1244/1393/3952/5020/7040 |
| biological process | GO:0007568 | aging | 1.68E-05 | 183/596/657/847/3952/4780/5580/5728/7040 |
| biological process | GO:0043271 | negative regulation of ion transport | 1.91E-05 | 477/596/805/845/3952/5728/7040 |
| biological process | GO:0010523 | negative regulation of calcium ion transport into cytosol | 1.91E-05 | 596/805/845/7040 |
| biological process | GO:0032930 | positive regulation of superoxide anion generation | 1.91E-05 | 183/2950/5580/7040 |
| biological process | GO:0031960 | response to corticosteroid | 2.23E-05 | 142/596/1244/2950/5020/7040/9370 |
| biological process | GO:2000379 | positive regulation of reactive oxygen species metabolic process | 2.81E-05 | 183/2950/3952/4780/5580/7040 |
| biological process | GO:0032928 | regulation of superoxide anion generation | 3.12E-05 | 183/2950/5580/7040 |
| biological process | GO:0001666 | response to hypoxia | 3.12E-05 | 596/847/1909/3952/4780/5465/5728/7040/9370 |
| biological process | GO:0035690 | cellular response to drug | 3.72E-05 | 196/845/1244/1393/4780/5580/5728/7040/9370 |
| biological process | GO:0036293 | response to decreased oxygen levels | 3.72E-05 | 596/847/1909/3952/4780/5465/5728/7040/9370 |
| biological process | GO:0031667 | response to nutrient levels | 4.42E-05 | 596/847/2950/3952/4780/5020/5465/5728/7040/9370 |
| biological process | GO:0048545 | response to steroid hormone | 4.83E-05 | 142/477/596/1244/2950/5020/5465/7040/9370 |
| biological process | GO:0072593 | reactive oxygen species metabolic process | 5.34E-05 | 183/596/847/2950/3952/4780/5580/7040 |
| biological process | GO:0070482 | response to oxygen levels | 5.40E-05 | 596/847/1909/3952/4780/5465/5728/7040/9370 |
| biological process | GO:2000377 | regulation of reactive oxygen species metabolic process | 5.40E-05 | 183/596/2950/3952/4780/5580/7040 |
| biological process | GO:0051926 | negative regulation of calcium ion transport | 6.04E-05 | 477/596/805/845/7040 |
| biological process | GO:0045471 | response to ethanol | 6.23E-05 | 847/1393/2950/3952/5728/9370 |
| biological process | GO:2001233 | regulation of apoptotic signaling pathway | 6.23E-05 | 142/183/596/637/1280/2950/4780/5580/5728 |
| biological process | GO:0070661 | leukocyte proliferation | 6.39E-05 | 196/596/977/1636/2950/3952/5580/7040 |
| biological process | GO:0032368 | regulation of lipid transport | 6.39E-05 | 183/3952/5020/5465/5580/9370 |
| biological process | GO:0034763 | negative regulation of transmembrane transport | 6.51E-05 | 477/805/845/3952/5728/7040 |
| biological process | GO:0006801 | superoxide metabolic process | 8.48E-05 | 183/2950/4780/5580/7040 |
| biological process | GO:0007204 | positive regulation of cytosolic calcium ion concentration | 9.58E-05 | 183/477/596/805/845/1909/5020/7040 |
| biological process | GO:0042310 | vasoconstriction | 9.86E-05 | 183/477/1636/1909/3952 |
| biological process | GO:0097191 | extrinsic apoptotic signaling pathway | 0.000104 | 183/596/637/1280/2950/5728/7040 |
| biological process | GO:0030534 | adult behavior | 0.000112 | 434/477/1393/3952/5465/5728 |
| biological process | GO:0001655 | urogenital system development | 0.000112 | 183/596/847/1286/1636/5728/7040/9370 |
| biological process | GO:0009743 | response to carbohydrate | 0.000116 | 847/2950/3952/5020/5728/7040/9370 |
| biological process | GO:0051384 | response to glucocorticoid | 0.000116 | 596/1244/2950/5020/7040/9370 |
| biological process | GO:0010742 | macrophage derived foam cell differentiation | 0.00012 | 183/5465/7040/9370 |
| biological process | GO:0042554 | superoxide anion generation | 0.00012 | 183/2950/5580/7040 |
| biological process | GO:0090077 | foam cell differentiation | 0.00012 | 183/5465/7040/9370 |
| biological process | GO:0014074 | response to purine-containing compound | 0.00012 | 196/845/1393/5020/5728/9370 |
| biological process | GO:0030258 | lipid modification | 0.000124 | 28/183/224/3952/5465/5728/9370 |
| biological process | GO:0014912 | negative regulation of smooth muscle cell migration | 0.000124 | 657/2950/4780/9370 |
| biological process | GO:0097756 | negative regulation of blood vessel diameter | 0.000124 | 183/477/1636/1909/3952 |
| biological process | GO:0003012 | muscle system process | 0.000124 | 142/183/477/805/845/1909/3952/5020/5465 |
| biological process | GO:1904705 | regulation of vascular smooth muscle cell proliferation | 0.000126 | 183/657/2950/5728/9370 |
| biological process | GO:1990874 | vascular smooth muscle cell proliferation | 0.000126 | 183/657/2950/5728/9370 |
| biological process | GO:2001236 | regulation of extrinsic apoptotic signaling pathway | 0.000133 | 183/596/637/1280/2950/5728 |
| biological process | GO:1905952 | regulation of lipid localization | 0.000141 | 183/3952/5020/5465/5580/9370 |
| biological process | GO:0051480 | regulation of cytosolic calcium ion concentration | 0.00015 | 183/477/596/805/845/1909/5020/7040 |
| biological process | GO:0060391 | positive regulation of SMAD protein signal transduction | 0.000159 | 142/657/7040 |
| biological process | GO:1904063 | negative regulation of cation transmembrane transport | 0.000162 | 477/805/845/5728/7040 |
| biological process | GO:1903170 | negative regulation of calcium ion transmembrane transport | 0.000162 | 477/805/845/7040 |
| biological process | GO:0006869 | lipid transport | 0.000166 | 183/1244/1636/3952/5020/5465/5580/9370 |
| biological process | GO:0090257 | regulation of muscle system process | 0.000179 | 142/183/477/805/845/5020/5465 |
| biological process | GO:0008631 | intrinsic apoptotic signaling pathway in response to oxidative stress | 0.000185 | 142/596/4780/5580 |
| biological process | GO:0044070 | regulation of anion transport | 0.000185 | 183/477/3952/5020/5580 |
| biological process | GO:0015908 | fatty acid transport | 0.000202 | 1244/1636/3952/5020/5465 |
| biological process | GO:0003018 | vascular process in circulatory system | 0.00021 | 183/477/1636/1909/3952/7040 |
| biological process | GO:0032943 | mononuclear cell proliferation | 0.00024 | 196/596/977/1636/3952/5580/7040 |
| biological process | GO:0010522 | regulation of calcium ion transport into cytosol | 0.000248 | 477/596/805/845/7040 |
| biological process | GO:0071466 | cellular response to xenobiotic stimulus | 0.000249 | 196/845/1244/1393/2950/7040 |
| biological process | GO:0034766 | negative regulation of ion transmembrane transport | 0.000249 | 477/805/845/5728/7040 |
| biological process | GO:0001822 | kidney development | 0.000249 | 183/596/847/1286/1636/7040/9370 |
| biological process | GO:0051280 | negative regulation of release of sequestered calcium ion into cytosol | 0.000262 | 805/845/7040 |
| biological process | GO:0001503 | ossification | 0.000262 | 596/657/847/1280/1462/3952/5020/7040 |
| biological process | GO:0010876 | lipid localization | 0.000262 | 183/1244/1636/3952/5020/5465/5580/9370 |
| biological process | GO:0032409 | regulation of transporter activity | 0.000262 | 477/596/805/845/1393/5580/5728 |
| biological process | GO:0071715 | icosanoid transport | 0.000262 | 1244/1636/3952/5020 |
| biological process | GO:1901571 | fatty acid derivative transport | 0.000262 | 1244/1636/3952/5020 |
| biological process | GO:0009410 | response to xenobiotic stimulus | 0.000313 | 196/845/1244/1393/2950/5020/7040 |
| biological process | GO:0072001 | renal system development | 0.000316 | 183/596/847/1286/1636/7040/9370 |
| biological process | GO:1903522 | regulation of blood circulation | 0.000341 | 183/477/805/845/1636/3952/5020 |
| biological process | GO:0015732 | prostaglandin transport | 0.000348 | 1244/3952/5020 |
| biological process | GO:0051284 | positive regulation of sequestering of calcium ion | 0.000348 | 805/845/7040 |
| biological process | GO:0035265 | organ growth | 0.000431 | 183/596/657/3952/5465/5728 |
| biological process | GO:0034284 | response to monosaccharide | 0.000463 | 847/2950/3952/5728/7040/9370 |
| biological process | GO:0019229 | regulation of vasoconstriction | 0.000469 | 183/477/1636/3952 |
| biological process | GO:0032890 | regulation of organic acid transport | 0.000496 | 183/477/3952/5020 |
| biological process | GO:0044282 | small molecule catabolic process | 0.000497 | 36/216/224/3952/5465/5728/7040/9370 |
| biological process | GO:0006979 | response to oxidative stress | 0.000541 | 142/596/847/1244/2950/4780/5580/9370 |
| biological process | GO:0006874 | cellular calcium ion homeostasis | 0.000595 | 183/477/596/805/845/1909/5020/7040 |
| biological process | GO:0007584 | response to nutrient | 0.000595 | 847/2950/3952/5728/7040/9370 |
| biological process | GO:0098754 | detoxification | 0.000617 | 847/1244/2950/2952/4780 |
| biological process | GO:0015849 | organic acid transport | 0.000617 | 183/477/1244/1636/3952/5020/5465 |
| biological process | GO:0046942 | carboxylic acid transport | 0.000617 | 183/477/1244/1636/3952/5020/5465 |
| biological process | GO:0030336 | negative regulation of cell migration | 0.000622 | 596/657/2950/4780/5728/7040/9370 |
| biological process | GO:0042326 | negative regulation of phosphorylation | 0.000651 | 259/805/2950/5465/5580/5728/7040/9370 |
| biological process | GO:0046683 | response to organophosphorus | 0.000651 | 196/1393/5020/5728/9370 |
| biological process | GO:0050765 | negative regulation of phagocytosis | 0.000651 | 5728/7040/9370 |
| biological process | GO:0060390 | regulation of SMAD protein signal transduction | 0.000651 | 142/657/7040 |
| biological process | GO:0071496 | cellular response to external stimulus | 0.000651 | 183/477/596/2950/3952/4780/7040 |
| biological process | GO:0055074 | calcium ion homeostasis | 0.000652 | 183/477/596/805/845/1909/5020/7040 |
| biological process | GO:1904062 | regulation of cation transmembrane transport | 0.000675 | 183/477/805/845/1393/5728/7040 |
| biological process | GO:0019748 | secondary metabolic process | 0.000696 | 434/596/1244/4780 |
| biological process | GO:0015711 | organic anion transport | 0.000747 | 183/477/1244/1636/3952/5020/5465/5580 |
| biological process | GO:2000146 | negative regulation of cell motility | 0.000747 | 596/657/2950/4780/5728/7040/9370 |
| biological process | GO:0072073 | kidney epithelium development | 0.000754 | 183/596/847/7040/9370 |
| biological process | GO:0010881 | regulation of cardiac muscle contraction by regulation of the release of sequestered calcium ion | 0.000792 | 477/805/845 |
| biological process | GO:0072006 | nephron development | 0.000792 | 183/596/1286/7040/9370 |
| biological process | GO:0035296 | regulation of tube diameter | 0.000798 | 183/477/1636/1909/3952 |
| biological process | GO:0050880 | regulation of blood vessel size | 0.000798 | 183/477/1636/1909/3952 |
| biological process | GO:0097746 | regulation of blood vessel diameter | 0.000798 | 183/477/1636/1909/3952 |
| biological process | GO:0072503 | cellular divalent inorganic cation homeostasis | 0.00081 | 183/477/596/805/845/1909/5020/7040 |
| biological process | GO:0035150 | regulation of tube size | 0.00081 | 183/477/1636/1909/3952 |
| biological process | GO:1904385 | cellular response to angiotensin | 0.000848 | 183/4780/5580 |
| biological process | GO:0045834 | positive regulation of lipid metabolic process | 0.000851 | 183/5465/5580/7040/9370 |
| biological process | GO:1903779 | regulation of cardiac conduction | 0.000869 | 183/477/805/845 |
| biological process | GO:1900182 | positive regulation of protein localization to nucleus | 0.000904 | 142/3952/5580/7040 |
| biological process | GO:0097237 | cellular response to toxic substance | 0.000904 | 847/2950/2952/4780/5580/5728 |
| biological process | GO:0071383 | cellular response to steroid hormone stimulus | 0.000959 | 142/477/1244/2950/5465/7040 |
| biological process | GO:0016202 | regulation of striated muscle tissue development | 0.000989 | 596/657/5465/5728/7040 |
| biological process | GO:0051098 | regulation of binding | 0.00099 | 142/596/1636/5465/5580/7040/9370 |
| biological process | GO:0051924 | regulation of calcium ion transport | 0.001022 | 183/477/596/805/845/7040 |
| biological process | GO:0055067 | monovalent inorganic cation homeostasis | 0.001027 | 183/477/533/596/5020 |
| biological process | GO:1901861 | regulation of muscle tissue development | 0.001031 | 596/657/5465/5728/7040 |
| biological process | GO:0050730 | regulation of peptidyl-tyrosine phosphorylation | 0.001031 | 183/1636/3952/5580/7040/9370 |
| biological process | GO:0010827 | regulation of glucose transmembrane transport | 0.001031 | 1909/3952/4780/9370 |
| biological process | GO:0032413 | negative regulation of ion transmembrane transporter activity | 0.001031 | 477/805/845/5728 |
| biological process | GO:0048634 | regulation of muscle organ development | 0.00105 | 596/657/5465/5728/7040 |
| biological process | GO:1990776 | response to angiotensin | 0.001063 | 183/4780/5580 |
| biological process | GO:0060402 | calcium ion transport into cytosol | 0.001098 | 477/596/805/845/7040 |
| biological process | GO:0051271 | negative regulation of cellular component movement | 0.0011 | 596/657/2950/4780/5728/7040/9370 |
| biological process | GO:0010644 | cell communication by electrical coupling | 0.00114 | 477/805/845 |
| biological process | GO:0010882 | regulation of cardiac muscle contraction by calcium ion signaling | 0.00114 | 477/805/845 |
| biological process | GO:0033032 | regulation of myeloid cell apoptotic process | 0.00114 | 596/5728/9370 |
| biological process | GO:0051279 | regulation of release of sequestered calcium ion into cytosol | 0.001181 | 477/805/845/7040 |
| biological process | GO:0015718 | monocarboxylic acid transport | 0.001183 | 1244/1636/3952/5020/5465 |
| biological process | GO:0022898 | regulation of transmembrane transporter activity | 0.00122 | 477/596/805/845/1393/5728 |
| biological process | GO:0010743 | regulation of macrophage derived foam cell differentiation | 0.00122 | 183/5465/9370 |
| biological process | GO:0010959 | regulation of metal ion transport | 0.00122 | 183/477/596/805/845/5728/7040 |
| biological process | GO:0040013 | negative regulation of locomotion | 0.001243 | 596/657/2950/4780/5728/7040/9370 |
| biological process | GO:0050886 | endocrine process | 0.001243 | 183/1393/1636/3952 |
| biological process | GO:0046651 | lymphocyte proliferation | 0.001286 | 196/596/977/3952/5580/7040 |
| biological process | GO:0016054 | organic acid catabolic process | 0.001347 | 36/224/3952/5465/7040/9370 |
| biological process | GO:0046395 | carboxylic acid catabolic process | 0.001347 | 36/224/3952/5465/7040/9370 |
| biological process | GO:0048660 | regulation of smooth muscle cell proliferation | 0.001357 | 183/657/2950/5728/9370 |
| biological process | GO:0010880 | regulation of release of sequestered calcium ion into cytosol by sarcoplasmic reticulum | 0.001406 | 477/805/845 |
| biological process | GO:0048659 | smooth muscle cell proliferation | 0.001406 | 183/657/2950/5728/9370 |
| biological process | GO:0060401 | cytosolic calcium ion transport | 0.001406 | 477/596/805/845/7040 |
| biological process | GO:0060047 | heart contraction | 0.001439 | 183/477/805/845/1636/5020 |
| biological process | GO:0032410 | negative regulation of transporter activity | 0.001466 | 477/805/845/5728 |
| biological process | GO:0033028 | myeloid cell apoptotic process | 0.001491 | 596/5728/9370 |
| biological process | GO:0051955 | regulation of amino acid transport | 0.001491 | 183/477/3952 |
| biological process | GO:2001235 | positive regulation of apoptotic signaling pathway | 0.001662 | 183/596/637/5580/5728 |
| biological process | GO:0003015 | heart process | 0.001662 | 183/477/805/845/1636/5020 |
| biological process | GO:0034330 | cell junction organization | 0.001662 | 183/596/977/1636/5728/7040 |
| biological process | GO:0033273 | response to vitamin | 0.001662 | 847/2950/3952/7040 |
| biological process | GO:0036473 | cell death in response to oxidative stress | 0.001662 | 142/596/4780/5580 |
| biological process | GO:0098801 | regulation of renal system process | 0.001714 | 183/5020/9370 |
| biological process | GO:0051952 | regulation of amine transport | 0.001783 | 183/477/3952/5020 |
| biological process | GO:0001933 | negative regulation of protein phosphorylation | 0.001806 | 259/805/2950/5580/5728/7040/9370 |
| biological process | GO:0043409 | negative regulation of MAPK cascade | 0.00181 | 259/2950/5580/5728/9370 |
| biological process | GO:0014808 | release of sequestered calcium ion into cytosol by sarcoplasmic reticulum | 0.00181 | 477/805/845 |
| biological process | GO:1901020 | negative regulation of calcium ion transmembrane transporter activity | 0.00181 | 477/805/845 |
| biological process | GO:0001657 | ureteric bud development | 0.001852 | 183/596/847/7040 |
| biological process | GO:0043255 | regulation of carbohydrate biosynthetic process | 0.001852 | 3952/5465/7040/9370 |
| biological process | GO:0051591 | response to cAMP | 0.001852 | 196/1393/5020/9370 |
| biological process | GO:0072163 | mesonephric epithelium development | 0.001875 | 183/596/847/7040 |
| biological process | GO:0072164 | mesonephric tubule development | 0.001875 | 183/596/847/7040 |
| biological process | GO:0042307 | positive regulation of protein import into nucleus | 0.001875 | 3952/5580/7040 |
| biological process | GO:0045777 | positive regulation of blood pressure | 0.001875 | 183/1636/5020 |
| biological process | GO:1903514 | release of sequestered calcium ion into cytosol by endoplasmic reticulum | 0.001875 | 477/805/845 |
| biological process | GO:0034599 | cellular response to oxidative stress | 0.001889 | 142/596/847/2950/4780/5580 |
| biological process | GO:0019395 | fatty acid oxidation | 0.001924 | 224/3952/5465/9370 |
| biological process | GO:0055024 | regulation of cardiac muscle tissue development | 0.001974 | 657/5465/5728/7040 |
| biological process | GO:0032094 | response to food | 0.001974 | 3952/5020/5465 |
| biological process | GO:1904706 | negative regulation of vascular smooth muscle cell proliferation | 0.001974 | 2950/5728/9370 |
| biological process | GO:0050731 | positive regulation of peptidyl-tyrosine phosphorylation | 0.001999 | 183/1636/3952/7040/9370 |
| biological process | GO:0002027 | regulation of heart rate | 0.001999 | 183/805/845/5020 |
| biological process | GO:0007631 | feeding behavior | 0.001999 | 183/434/3952/5020 |
| biological process | GO:0034440 | lipid oxidation | 0.001999 | 224/3952/5465/9370 |
| biological process | GO:1901654 | response to ketone | 0.002032 | 142/196/1244/5020/7040 |
| biological process | GO:0001823 | mesonephros development | 0.002032 | 183/596/847/7040 |
| biological process | GO:0015837 | amine transport | 0.002032 | 183/477/3952/5020 |
| biological process | GO:0098869 | cellular oxidant detoxification | 0.002032 | 847/2950/2952/4780 |
| biological process | GO:1904591 | positive regulation of protein import | 0.002032 | 3952/5580/7040 |
| biological process | GO:0009314 | response to radiation | 0.002035 | 142/477/596/847/1153/2189/7040 |
| biological process | GO:0032091 | negative regulation of protein binding | 0.002076 | 1636/5465/5580/9370 |
| biological process | GO:0090184 | positive regulation of kidney development | 0.002158 | 183/7040/9370 |
| biological process | GO:0055017 | cardiac muscle tissue growth | 0.002213 | 183/657/5465/5728 |
| biological process | GO:0003300 | cardiac muscle hypertrophy | 0.002283 | 142/183/3952/5465 |
| biological process | GO:1902893 | regulation of pri-miRNA transcription by RNA polymerase II | 0.002288 | 657/5465/7040 |
| biological process | GO:0062014 | negative regulation of small molecule metabolic process | 0.002343 | 142/5465/7040/9370 |
| biological process | GO:0009062 | fatty acid catabolic process | 0.002391 | 224/3952/5465/9370 |
| biological process | GO:0030004 | cellular monovalent inorganic cation homeostasis | 0.002391 | 183/477/533/596 |
| biological process | GO:1904659 | glucose transmembrane transport | 0.002391 | 1909/3952/4780/9370 |
| biological process | GO:1903829 | positive regulation of cellular protein localization | 0.002433 | 142/596/637/3952/5580/7040 |
| biological process | GO:0014897 | striated muscle hypertrophy | 0.00244 | 142/183/3952/5465 |
| biological process | GO:0072009 | nephron epithelium development | 0.00244 | 183/596/7040/9370 |
| biological process | GO:0006939 | smooth muscle contraction | 0.002515 | 183/477/1909/5020 |
| biological process | GO:0070296 | sarcoplasmic reticulum calcium ion transport | 0.002521 | 477/805/845 |
| biological process | GO:0014896 | muscle hypertrophy | 0.002572 | 142/183/3952/5465 |
| biological process | GO:0007623 | circadian rhythm | 0.002572 | 196/3952/5465/5728/9370 |
| biological process | GO:0051235 | maintenance of location | 0.002572 | 477/805/845/3952/5465/7040 |
| biological process | GO:0008645 | hexose transmembrane transport | 0.002572 | 1909/3952/4780/9370 |
| biological process | GO:0060419 | heart growth | 0.002572 | 183/657/5465/5728 |
| biological process | GO:1990748 | cellular detoxification | 0.002572 | 847/2950/2952/4780 |
| biological process | GO:0014003 | oligodendrocyte development | 0.002572 | 2950/5728/7040 |
| biological process | GO:0030225 | macrophage differentiation | 0.002572 | 142/7040/9370 |
| biological process | GO:0035307 | positive regulation of protein dephosphorylation | 0.002572 | 805/5580/7040 |
| biological process | GO:0044060 | regulation of endocrine process | 0.002572 | 183/1393/3952 |
| biological process | GO:0015749 | monosaccharide transmembrane transport | 0.002712 | 1909/3952/4780/9370 |
| biological process | GO:0001974 | blood vessel remodeling | 0.002712 | 183/1636/7040 |
| biological process | GO:0032309 | icosanoid secretion | 0.002712 | 1636/3952/5020 |
| biological process | GO:0034765 | regulation of ion transmembrane transport | 0.002805 | 183/477/805/845/1393/5728/7040 |
| biological process | GO:0007569 | cell aging | 0.002805 | 596/657/5580/5728 |
| biological process | GO:0034219 | carbohydrate transmembrane transport | 0.002805 | 1909/3952/4780/9370 |
| biological process | GO:1900180 | regulation of protein localization to nucleus | 0.002805 | 142/3952/5580/7040 |
| biological process | GO:0045776 | negative regulation of blood pressure | 0.002805 | 5020/5465/9370 |
| biological process | GO:1904738 | vascular associated smooth muscle cell migration | 0.002805 | 183/4780/9370 |
| biological process | GO:1904752 | regulation of vascular associated smooth muscle cell migration | 0.002805 | 183/4780/9370 |
| biological process | GO:0043393 | regulation of protein binding | 0.002866 | 596/1636/5465/5580/9370 |
| biological process | GO:0044242 | cellular lipid catabolic process | 0.002866 | 224/3952/5465/5580/9370 |
| biological process | GO:0072511 | divalent inorganic cation transport | 0.002898 | 183/477/596/805/845/1244/7040 |
| biological process | GO:0001101 | response to acid chemical | 0.002898 | 847/1244/2950/3952/5020/9370 |
| biological process | GO:0031952 | regulation of protein autophosphorylation | 0.002899 | 805/1636/9370 |
| biological process | GO:0060986 | endocrine hormone secretion | 0.002899 | 183/1393/3952 |
| biological process | GO:0061614 | pri-miRNA transcription by RNA polymerase II | 0.002899 | 657/5465/7040 |
| biological process | GO:0048762 | mesenchymal cell differentiation | 0.002912 | 596/657/1909/5728/7040 |
| biological process | GO:0003014 | renal system process | 0.003033 | 183/596/5020/9370 |
| biological process | GO:0006111 | regulation of gluconeogenesis | 0.003033 | 3952/5465/9370 |
| biological process | GO:0045912 | negative regulation of carbohydrate metabolic process | 0.003033 | 5465/7040/9370 |
| biological process | GO:0070663 | regulation of leukocyte proliferation | 0.003047 | 196/596/2950/3952/7040 |
| biological process | GO:0035094 | response to nicotine | 0.003184 | 477/596/5465 |
| biological process | GO:2001238 | positive regulation of extrinsic apoptotic signaling pathway | 0.003184 | 183/637/5728 |
| biological process | GO:1903793 | positive regulation of anion transport | 0.00329 | 183/5020/5580 |
| biological process | GO:0008343 | adult feeding behavior | 0.00329 | 434/3952 |
| biological process | GO:0042756 | drinking behavior | 0.00329 | 183/5020 |
| biological process | GO:1903789 | regulation of amino acid transmembrane transport | 0.00329 | 183/477 |
| biological process | GO:0014066 | regulation of phosphatidylinositol 3-kinase signaling | 0.00329 | 183/847/3952/5728 |
| biological process | GO:0046717 | acid secretion | 0.00329 | 1244/1636/3952/5020 |
| biological process | GO:0051209 | release of sequestered calcium ion into cytosol | 0.00329 | 477/805/845/7040 |
| biological process | GO:2001234 | negative regulation of apoptotic signaling pathway | 0.00344 | 596/637/1280/2950/4780 |
| biological process | GO:0006936 | muscle contraction | 0.00344 | 183/477/805/845/1909/5020 |
| biological process | GO:0018107 | peptidyl-threonine phosphorylation | 0.00344 | 596/805/5580/7040 |
| biological process | GO:0051283 | negative regulation of sequestering of calcium ion | 0.00344 | 477/805/845/7040 |
| biological process | GO:0000302 | response to reactive oxygen species | 0.003508 | 596/847/2950/4780/5580 |
| biological process | GO:0042445 | hormone metabolic process | 0.003508 | 216/220/1586/1636/3952 |
| biological process | GO:0018108 | peptidyl-tyrosine phosphorylation | 0.003548 | 183/1636/3952/5580/7040/9370 |
| biological process | GO:0048738 | cardiac muscle tissue development | 0.003549 | 183/657/5465/5728/7040 |
| biological process | GO:0055078 | sodium ion homeostasis | 0.003552 | 183/477/5020 |
| biological process | GO:0006066 | alcohol metabolic process | 0.003552 | 216/220/224/847/3952/5728 |
| biological process | GO:0051282 | regulation of sequestering of calcium ion | 0.003552 | 477/805/845/7040 |
| biological process | GO:0032102 | negative regulation of response to external stimulus | 0.003582 | 2950/3952/5465/5580/5728/9370 |
| biological process | GO:0018212 | peptidyl-tyrosine modification | 0.00362 | 183/1636/3952/5580/7040/9370 |
| biological process | GO:0071320 | cellular response to cAMP | 0.00369 | 196/1393/9370 |
| biological process | GO:0014820 | tonic smooth muscle contraction | 0.003719 | 183/1909 |
| biological process | GO:0016264 | gap junction assembly | 0.003719 | 183/1636 |
| biological process | GO:0060315 | negative regulation of ryanodine-sensitive calcium-release channel activity | 0.003719 | 805/845 |
| biological process | GO:0070471 | uterine smooth muscle contraction | 0.003719 | 183/5020 |
| biological process | GO:1901201 | regulation of extracellular matrix assembly | 0.003719 | 183/7040 |
| biological process | GO:0051208 | sequestering of calcium ion | 0.003746 | 477/805/845/7040 |
| biological process | GO:0033002 | muscle cell proliferation | 0.003792 | 183/657/2950/5728/9370 |
| biological process | GO:0072329 | monocarboxylic acid catabolic process | 0.003826 | 224/3952/5465/9370 |
| biological process | GO:0034329 | cell junction assembly | 0.003909 | 183/596/977/1636/5728 |
| biological process | GO:0042306 | regulation of protein import into nucleus | 0.003962 | 3952/5580/7040 |
| biological process | GO:0018210 | peptidyl-threonine modification | 0.004005 | 596/805/5580/7040 |
| biological process | GO:0006749 | glutathione metabolic process | 0.004117 | 2950/2952/4780 |
| biological process | GO:0086065 | cell communication involved in cardiac conduction | 0.004117 | 477/805/845 |
| biological process | GO:0090183 | regulation of kidney development | 0.004117 | 183/7040/9370 |
| biological process | GO:0010649 | regulation of cell communication by electrical coupling | 0.004275 | 805/845 |
| biological process | GO:0033197 | response to vitamin E | 0.004275 | 847/3952 |
| biological process | GO:0010665 | regulation of cardiac muscle cell apoptotic process | 0.004289 | 183/4780/5728 |
| biological process | GO:0006631 | fatty acid metabolic process | 0.004289 | 36/224/2950/3952/5465/9370 |
| biological process | GO:0045926 | negative regulation of growth | 0.004374 | 183/596/5465/5728/7040 |
| biological process | GO:0035306 | positive regulation of dephosphorylation | 0.004435 | 805/5580/7040 |
| biological process | GO:0071385 | cellular response to glucocorticoid stimulus | 0.004435 | 1244/2950/7040 |
| biological process | GO:1904589 | regulation of protein import | 0.004435 | 3952/5580/7040 |
| biological process | GO:0008016 | regulation of heart contraction | 0.004471 | 183/477/805/845/5020 |
| biological process | GO:0097553 | calcium ion transmembrane import into cytosol | 0.004509 | 477/805/845/7040 |
| biological process | GO:0014706 | striated muscle tissue development | 0.004534 | 183/596/657/5465/5728/7040 |
| biological process | GO:0001658 | branching involved in ureteric bud morphogenesis | 0.004534 | 183/596/7040 |
| biological process | GO:0010662 | regulation of striated muscle cell apoptotic process | 0.004534 | 183/4780/5728 |
| biological process | GO:0045843 | negative regulation of striated muscle tissue development | 0.004534 | 5465/5728/7040 |
| biological process | GO:0008286 | insulin receptor signaling pathway | 0.004534 | 183/533/3952/5580 |
| biological process | GO:0009411 | response to UV | 0.004534 | 142/596/847/1153 |
| biological process | GO:0010659 | cardiac muscle cell apoptotic process | 0.004573 | 183/4780/5728 |
| biological process | GO:0046324 | regulation of glucose import | 0.004573 | 3952/4780/9370 |
| biological process | GO:0048635 | negative regulation of muscle organ development | 0.004573 | 5465/5728/7040 |
| biological process | GO:0007611 | learning or memory | 0.004573 | 183/477/1393/5020/5728 |
| biological process | GO:0006606 | protein import into nucleus | 0.004573 | 183/3952/5580/7040 |
| biological process | GO:0106106 | cold-induced thermogenesis | 0.004573 | 216/3952/5020/9370 |
| biological process | GO:0120161 | regulation of cold-induced thermogenesis | 0.004573 | 216/3952/5020/9370 |
| biological process | GO:0005513 | detection of calcium ion | 0.004573 | 805/845 |
| biological process | GO:0007183 | SMAD protein complex assembly | 0.004573 | 142/7040 |
| biological process | GO:0010745 | negative regulation of macrophage derived foam cell differentiation | 0.004573 | 5465/9370 |
| biological process | GO:0032096 | negative regulation of response to food | 0.004573 | 3952/5465 |
| biological process | GO:0032099 | negative regulation of appetite | 0.004573 | 3952/5465 |
| biological process | GO:0042711 | maternal behavior | 0.004573 | 5020/5728 |
| biological process | GO:0060746 | parental behavior | 0.004573 | 5020/5728 |
| biological process | GO:0032370 | positive regulation of lipid transport | 0.004736 | 5020/5580/9370 |
| biological process | GO:0071384 | cellular response to corticosteroid stimulus | 0.004736 | 1244/2950/7040 |
| biological process | GO:0071901 | negative regulation of protein serine/threonine kinase activity | 0.004757 | 2950/5580/5728/9370 |
| biological process | GO:0032412 | regulation of ion transmembrane transporter activity | 0.004817 | 477/805/845/1393/5728 |
| biological process | GO:0042542 | response to hydrogen peroxide | 0.004824 | 596/847/4780/5580 |
| biological process | GO:0061337 | cardiac conduction | 0.004824 | 183/477/805/845 |
| biological process | GO:0010658 | striated muscle cell apoptotic process | 0.004824 | 183/4780/5728 |
| biological process | GO:0032835 | glomerulus development | 0.004824 | 596/1286/9370 |
| biological process | GO:0046824 | positive regulation of nucleocytoplasmic transport | 0.004824 | 3952/5580/7040 |
| biological process | GO:0048857 | neural nucleus development | 0.004824 | 220/596/805 |
| biological process | GO:1901862 | negative regulation of muscle tissue development | 0.004824 | 5465/5728/7040 |
| biological process | GO:0015850 | organic hydroxy compound transport | 0.004833 | 183/1244/3952/5020/9370 |
| biological process | GO:0034504 | protein localization to nucleus | 0.004833 | 142/183/3952/5580/7040 |
| biological process | GO:0071236 | cellular response to antibiotic | 0.004833 | 196/4780/5580/5728 |
| biological process | GO:0008643 | carbohydrate transport | 0.004927 | 1909/3952/4780/9370 |
| biological process | GO:0014065 | phosphatidylinositol 3-kinase signaling | 0.004927 | 183/847/3952/5728 |
| biological process | GO:0031330 | negative regulation of cellular catabolic process | 0.004951 | 596/819/1153/3952/5465 |
| biological process | GO:0032310 | prostaglandin secretion | 0.005009 | 3952/5020 |
| biological process | GO:0035815 | positive regulation of renal sodium excretion | 0.005009 | 183/5020 |
| biological process | GO:0048754 | branching morphogenesis of an epithelial tube | 0.005111 | 183/596/1909/7040 |
| biological process | GO:0060537 | muscle tissue development | 0.005111 | 183/596/657/5465/5728/7040 |
| biological process | GO:0016999 | antibiotic metabolic process | 0.005195 | 48/216/847/1244 |
| biological process | GO:1903169 | regulation of calcium ion transmembrane transport | 0.005195 | 477/805/845/7040 |
| biological process | GO:0019216 | regulation of lipid metabolic process | 0.005195 | 183/3952/5465/5580/7040/9370 |
| biological process | GO:0006026 | aminoglycan catabolic process | 0.005287 | 1462/1486/7040 |
| biological process | GO:0060675 | ureteric bud morphogenesis | 0.005287 | 183/596/7040 |
| biological process | GO:0007548 | sex differentiation | 0.005314 | 596/657/1586/2189/3952 |
| biological process | GO:0046626 | regulation of insulin receptor signaling pathway | 0.005461 | 183/3952/5580 |
| biological process | GO:0046888 | negative regulation of hormone secretion | 0.005461 | 1393/3952/9370 |
| biological process | GO:0072171 | mesonephric tubule morphogenesis | 0.005461 | 183/596/7040 |
| biological process | GO:1990845 | adaptive thermogenesis | 0.005461 | 216/3952/5020/9370 |
| biological process | GO:0032105 | negative regulation of response to extracellular stimulus | 0.005511 | 3952/5465 |
| biological process | GO:0032108 | negative regulation of response to nutrient levels | 0.005511 | 3952/5465 |
| biological process | GO:0034616 | response to laminar fluid shear stress | 0.005511 | 4780/7040 |
| biological process | GO:0048662 | negative regulation of smooth muscle cell proliferation | 0.005601 | 2950/5728/9370 |
| biological process | GO:0051705 | multi-organism behavior | 0.005601 | 1393/5020/5728 |
| biological process | GO:0043270 | positive regulation of ion transport | 0.005601 | 183/805/3952/5020/5580 |
| biological process | GO:0045806 | negative regulation of endocytosis | 0.005802 | 5728/7040/9370 |
| biological process | GO:0046323 | glucose import | 0.005802 | 3952/4780/9370 |
| biological process | GO:0060485 | mesenchyme development | 0.00583 | 596/657/1909/5728/7040 |
| biological process | GO:0032680 | regulation of tumor necrosis factor production | 0.006116 | 671/2950/3952/9370 |
| biological process | GO:0014061 | regulation of norepinephrine secretion | 0.006123 | 183/5020 |
| biological process | GO:2001170 | negative regulation of ATP biosynthetic process | 0.006123 | 142/5465 |
| biological process | GO:0032720 | negative regulation of tumor necrosis factor production | 0.006205 | 671/2950/9370 |
| biological process | GO:0060395 | SMAD protein signal transduction | 0.006205 | 142/657/7040 |
| biological process | GO:0042440 | pigment metabolic process | 0.006387 | 259/434/596 |
| biological process | GO:0031214 | biomineral tissue development | 0.006387 | 657/3952/5465/7040 |
| biological process | GO:0032640 | tumor necrosis factor production | 0.006387 | 671/2950/3952/9370 |
| biological process | GO:0051170 | import into nucleus | 0.006387 | 183/3952/5580/7040 |
| biological process | GO:1903555 | regulation of tumor necrosis factor superfamily cytokine production | 0.006387 | 671/2950/3952/9370 |
| biological process | GO:0006816 | calcium ion transport | 0.006435 | 183/477/596/805/845/7040 |
| biological process | GO:0042391 | regulation of membrane potential | 0.006435 | 142/477/596/637/845/5728 |
| biological process | GO:0006635 | fatty acid beta-oxidation | 0.006546 | 3952/5465/9370 |
| biological process | GO:0051881 | regulation of mitochondrial membrane potential | 0.006546 | 142/596/637 |
| biological process | GO:1903556 | negative regulation of tumor necrosis factor superfamily cytokine production | 0.006546 | 671/2950/9370 |
| biological process | GO:2001242 | regulation of intrinsic apoptotic signaling pathway | 0.006571 | 142/596/637/4780 |
| biological process | GO:0007162 | negative regulation of cell adhesion | 0.006592 | 5465/5580/5728/7040/9370 |
| biological process | GO:0097193 | intrinsic apoptotic signaling pathway | 0.006592 | 142/596/637/4780/5580 |
| biological process | GO:0006883 | cellular sodium ion homeostasis | 0.006601 | 183/477 |
| biological process | GO:0048243 | norepinephrine secretion | 0.006601 | 183/5020 |
| biological process | GO:0002244 | hematopoietic progenitor cell differentiation | 0.006628 | 596/1636/4780/7040 |
| biological process | GO:0071356 | cellular response to tumor necrosis factor | 0.006723 | 1244/1393/2950/4780/9370 |
| biological process | GO:0071706 | tumor necrosis factor superfamily cytokine production | 0.006839 | 671/2950/3952/9370 |
| biological process | GO:0051146 | striated muscle cell differentiation | 0.006839 | 183/596/845/5465/7040 |
| biological process | GO:0006081 | cellular aldehyde metabolic process | 0.006839 | 216/220/224 |
| biological process | GO:0070373 | negative regulation of ERK1 and ERK2 cascade | 0.006839 | 2950/5728/9370 |
| biological process | GO:0072078 | nephron tubule morphogenesis | 0.006839 | 183/596/7040 |
| biological process | GO:1900076 | regulation of cellular response to insulin stimulus | 0.006839 | 183/3952/5580 |
| biological process | GO:0050728 | negative regulation of inflammatory response | 0.006913 | 2950/5465/5580/9370 |
| biological process | GO:0051100 | negative regulation of binding | 0.006913 | 1636/5465/5580/9370 |
| biological process | GO:0048511 | rhythmic process | 0.006973 | 196/3952/5465/5728/9370 |
| biological process | GO:0010611 | regulation of cardiac muscle hypertrophy | 0.007031 | 142/183/5465 |
| biological process | GO:0050890 | cognition | 0.007031 | 183/477/1393/5020/5728 |
| biological process | GO:0030809 | negative regulation of nucleotide biosynthetic process | 0.007031 | 142/5465 |
| biological process | GO:0044320 | cellular response to leptin stimulus | 0.007031 | 3952/5728 |
| biological process | GO:0071605 | monocyte chemotactic protein-1 production | 0.007031 | 2950/9370 |
| biological process | GO:0071637 | regulation of monocyte chemotactic protein-1 production | 0.007031 | 2950/9370 |
| biological process | GO:0090190 | positive regulation of branching involved in ureteric bud morphogenesis | 0.007031 | 183/7040 |
| biological process | GO:1900372 | negative regulation of purine nucleotide biosynthetic process | 0.007031 | 142/5465 |
| biological process | GO:0006937 | regulation of muscle contraction | 0.007031 | 477/805/845/5020 |
| biological process | GO:0072088 | nephron epithelium morphogenesis | 0.007138 | 183/596/7040 |
| biological process | GO:0001659 | temperature homeostasis | 0.007284 | 216/3952/5020/9370 |
| biological process | GO:0055002 | striated muscle cell development | 0.007284 | 183/596/845/5465 |
| biological process | GO:0014743 | regulation of muscle hypertrophy | 0.007558 | 142/183/5465 |
| biological process | GO:0043407 | negative regulation of MAP kinase activity | 0.007558 | 2950/5580/9370 |
| biological process | GO:0055021 | regulation of cardiac muscle tissue growth | 0.007558 | 657/5465/5728 |
| biological process | GO:0061333 | renal tubule morphogenesis | 0.007558 | 183/596/7040 |
| biological process | GO:0072028 | nephron morphogenesis | 0.007558 | 183/596/7040 |
| biological process | GO:0010663 | positive regulation of striated muscle cell apoptotic process | 0.007598 | 183/5728 |
| biological process | GO:0010666 | positive regulation of cardiac muscle cell apoptotic process | 0.007598 | 183/5728 |
| biological process | GO:0010888 | negative regulation of lipid storage | 0.007598 | 3952/5465 |
| biological process | GO:0032095 | regulation of response to food | 0.007598 | 3952/5465 |
| biological process | GO:0046034 | ATP metabolic process | 0.007638 | 142/477/637/5465/7040 |
| biological process | GO:0071260 | cellular response to mechanical stimulus | 0.007722 | 183/477/7040 |
| biological process | GO:0009895 | negative regulation of catabolic process | 0.007937 | 596/819/1153/3952/5465 |
| biological process | GO:0048771 | tissue remodeling | 0.007993 | 183/1636/3952/7040 |
| biological process | GO:0022408 | negative regulation of cell-cell adhesion | 0.008287 | 5465/5580/7040/9370 |
| biological process | GO:0048015 | phosphatidylinositol-mediated signaling | 0.008287 | 183/847/3952/5728 |
| biological process | GO:0034612 | response to tumor necrosis factor | 0.008321 | 1244/1393/2950/4780/9370 |
| biological process | GO:0061138 | morphogenesis of a branching epithelium | 0.008389 | 183/596/1909/7040 |
| biological process | GO:0006855 | drug transmembrane transport | 0.008389 | 183/477/1244 |
| biological process | GO:0032637 | interleukin-8 production | 0.008389 | 671/3952/9370 |
| biological process | GO:0055117 | regulation of cardiac muscle contraction | 0.008389 | 477/805/845 |
| biological process | GO:1905954 | positive regulation of lipid localization | 0.008389 | 5020/5580/9370 |
| biological process | GO:0009416 | response to light stimulus | 0.008433 | 142/477/596/847/1153 |
| biological process | GO:0048017 | inositol lipid-mediated signaling | 0.008624 | 183/847/3952/5728 |
| biological process | GO:0048145 | regulation of fibroblast proliferation | 0.008624 | 183/2950/7040 |
| biological process | GO:0070371 | ERK1 and ERK2 cascade | 0.008725 | 183/2950/5728/7040/9370 |
| biological process | GO:0048144 | fibroblast proliferation | 0.008725 | 183/2950/7040 |
| biological process | GO:0060420 | regulation of heart growth | 0.008725 | 657/5465/5728 |
| biological process | GO:0015874 | norepinephrine transport | 0.008725 | 183/5020 |
| biological process | GO:0035813 | regulation of renal sodium excretion | 0.008725 | 183/5020 |
| biological process | GO:0051900 | regulation of mitochondrial depolarization | 0.008725 | 142/596 |
| biological process | GO:0061213 | positive regulation of mesonephros development | 0.008725 | 183/7040 |
| biological process | GO:1903055 | positive regulation of extracellular matrix organization | 0.008725 | 183/7040 |
| biological process | GO:1904753 | negative regulation of vascular associated smooth muscle cell migration | 0.008725 | 4780/9370 |
| biological process | GO:0030308 | negative regulation of cell growth | 0.008725 | 183/596/5465/7040 |
| biological process | GO:0055001 | muscle cell development | 0.008725 | 183/596/845/5465 |
| biological process | GO:0016311 | dephosphorylation | 0.008892 | 53/596/805/5580/5728/7040 |
| biological process | GO:0014031 | mesenchymal cell development | 0.008923 | 596/657/1909 |
| biological process | GO:0034308 | primary alcohol metabolic process | 0.008923 | 216/220/224 |
| biological process | GO:0006470 | protein dephosphorylation | 0.008923 | 596/805/5580/5728/7040 |
| biological process | GO:0009914 | hormone transport | 0.009025 | 183/1244/1393/3952/9370 |
| biological process | GO:0008625 | extrinsic apoptotic signaling pathway via death domain receptors | 0.009149 | 596/637/5728 |
| biological process | GO:0070542 | response to fatty acid | 0.009149 | 847/5020/9370 |
| biological process | GO:0070838 | divalent metal ion transport | 0.009157 | 183/477/596/805/845/7040 |
| biological process | GO:0006582 | melanin metabolic process | 0.009157 | 434/596 |
| biological process | GO:0010869 | regulation of receptor biosynthetic process | 0.009157 | 5465/9370 |
| biological process | GO:0044321 | response to leptin | 0.009157 | 3952/5728 |
| biological process | GO:0045649 | regulation of macrophage differentiation | 0.009157 | 7040/9370 |
| biological process | GO:0046628 | positive regulation of insulin receptor signaling pathway | 0.009157 | 183/3952 |
| biological process | GO:0090189 | regulation of branching involved in ureteric bud morphogenesis | 0.009157 | 183/7040 |
| biological process | GO:1901685 | glutathione derivative metabolic process | 0.009157 | 2950/2952 |
| biological process | GO:1901687 | glutathione derivative biosynthetic process | 0.009157 | 2950/2952 |
| biological process | GO:0048871 | multicellular organismal homeostasis | 0.009157 | 216/596/1280/3952/5020/9370 |
| biological process | GO:0050727 | regulation of inflammatory response | 0.009157 | 183/2950/3952/5465/5580/9370 |
| biological process | GO:0014068 | positive regulation of phosphatidylinositol 3-kinase signaling | 0.009157 | 183/847/3952 |
| biological process | GO:0045844 | positive regulation of striated muscle tissue development | 0.009157 | 596/657/7040 |
| biological process | GO:0048636 | positive regulation of muscle organ development | 0.009157 | 596/657/7040 |
| biological process | GO:0043112 | receptor metabolic process | 0.009273 | 819/5465/7040/9370 |
| biological process | GO:0044272 | sulfur compound biosynthetic process | 0.009273 | 1462/2950/2952/4780 |
| biological process | GO:1901136 | carbohydrate derivative catabolic process | 0.009273 | 1462/1486/5580/7040 |
| biological process | GO:0034103 | regulation of tissue remodeling | 0.009336 | 183/3952/7040 |
| biological process | GO:1901863 | positive regulation of muscle tissue development | 0.009336 | 596/657/7040 |
| biological process | GO:1901888 | regulation of cell junction assembly | 0.009336 | 183/1636/5728 |
| biological process | GO:0006094 | gluconeogenesis | 0.009601 | 3952/5465/9370 |
| biological process | GO:1901019 | regulation of calcium ion transmembrane transporter activity | 0.009601 | 477/805/845 |
| biological process | GO:0071214 | cellular response to abiotic stimulus | 0.009601 | 142/183/477/5728/7040 |
| biological process | GO:0104004 | cellular response to environmental stimulus | 0.009601 | 142/183/477/5728/7040 |
| biological process | GO:0007617 | mating behavior | 0.009621 | 5020/5728 |
| biological process | GO:0032098 | regulation of appetite | 0.009621 | 3952/5465 |
| biological process | GO:0035812 | renal sodium excretion | 0.009621 | 183/5020 |
| biological process | GO:0051882 | mitochondrial depolarization | 0.009621 | 142/596 |
| biological process | GO:0089718 | amino acid import across plasma membrane | 0.009621 | 183/477 |
| biological process | GO:1901889 | negative regulation of cell junction assembly | 0.009621 | 1636/5728 |
| biological process | GO:0001763 | morphogenesis of a branching structure | 0.009676 | 183/596/1909/7040 |
| biological process | GO:0016042 | lipid catabolic process | 0.009676 | 224/3952/5465/5580/9370 |
| biological process | GO:0045778 | positive regulation of ossification | 0.009676 | 657/5020/7040 |
| biological process | GO:0009205 | purine ribonucleoside triphosphate metabolic process | 0.0099 | 142/477/637/5465/7040 |
| biological process | GO:0051899 | membrane depolarization | 0.009945 | 142/477/596 |
| biological process | GO:0031099 | regeneration | 0.009952 | 596/2950/5728/7040 |
| biological process | GO:0002446 | neutrophil mediated immunity | 0.010036 | 671/820/847/1636/2950/5580 |
| biological process | GO:0017038 | protein import | 0.010094 | 183/3952/5580/7040 |
| biological process | GO:0019319 | hexose biosynthetic process | 0.010166 | 3952/5465/9370 |
| biological process | GO:0010804 | negative regulation of tumor necrosis factor-mediated signaling pathway | 0.010166 | 2950/9370 |
| biological process | GO:0044062 | regulation of excretion | 0.010166 | 183/5020 |
| biological process | GO:0050996 | positive regulation of lipid catabolic process | 0.010166 | 5465/5580 |
| biological process | GO:0086064 | cell communication by electrical coupling involved in cardiac conduction | 0.010166 | 477/805 |
| biological process | GO:1900078 | positive regulation of cellular response to insulin stimulus | 0.010166 | 183/3952 |
| biological process | GO:0009167 | purine ribonucleoside monophosphate metabolic process | 0.010315 | 142/477/637/5465/7040 |
| biological process | GO:0072080 | nephron tubule development | 0.010315 | 183/596/7040 |
| biological process | GO:1901655 | cellular response to ketone | 0.010315 | 196/1244/7040 |
| biological process | GO:0009126 | purine nucleoside monophosphate metabolic process | 0.010363 | 142/477/637/5465/7040 |
| biological process | GO:0009199 | ribonucleoside triphosphate metabolic process | 0.010363 | 142/477/637/5465/7040 |
| biological process | GO:0009144 | purine nucleoside triphosphate metabolic process | 0.010474 | 142/477/637/5465/7040 |
| biological process | GO:0060993 | kidney morphogenesis | 0.010536 | 183/596/7040 |
| biological process | GO:0030278 | regulation of ossification | 0.010536 | 596/657/5020/7040 |
| biological process | GO:0010721 | negative regulation of cell development | 0.010626 | 596/657/5465/5728/7040 |
| biological process | GO:0034764 | positive regulation of transmembrane transport | 0.010626 | 183/805/4780/9370 |
| biological process | GO:0035637 | multicellular organismal signaling | 0.010626 | 183/477/805/845 |
| biological process | GO:0014829 | vascular smooth muscle contraction | 0.010626 | 183/1909 |
| biological process | GO:0032104 | regulation of response to extracellular stimulus | 0.010626 | 3952/5465 |
| biological process | GO:0032107 | regulation of response to nutrient levels | 0.010626 | 3952/5465 |
| biological process | GO:0061217 | regulation of mesonephros development | 0.010626 | 183/7040 |
| biological process | GO:2000679 | positive regulation of transcription regulatory region DNA binding | 0.010626 | 142/7040 |
| biological process | GO:0042100 | B cell proliferation | 0.010626 | 196/596/5580 |
| biological process | GO:0061326 | renal tubule development | 0.010626 | 183/596/7040 |
| biological process | GO:0071222 | cellular response to lipopolysaccharide | 0.010674 | 671/1244/2950/7040 |
| biological process | GO:0048638 | regulation of developmental growth | 0.010821 | 596/657/3952/5465/5728 |
| biological process | GO:0006109 | regulation of carbohydrate metabolic process | 0.010821 | 3952/5465/7040/9370 |
| biological process | GO:0008585 | female gonad development | 0.010837 | 596/2189/3952 |
| biological process | GO:0010660 | regulation of muscle cell apoptotic process | 0.010837 | 183/4780/5728 |
| biological process | GO:0050670 | regulation of lymphocyte proliferation | 0.010994 | 196/596/3952/7040 |
| biological process | GO:0006942 | regulation of striated muscle contraction | 0.010994 | 477/805/845 |
| biological process | GO:0007044 | cell-substrate junction assembly | 0.010994 | 596/977/5728 |
| biological process | GO:0048709 | oligodendrocyte differentiation | 0.010994 | 2950/5728/7040 |
| biological process | GO:0120162 | positive regulation of cold-induced thermogenesis | 0.010994 | 3952/5020/9370 |
| biological process | GO:0032944 | regulation of mononuclear cell proliferation | 0.010994 | 196/596/3952/7040 |
| biological process | GO:0051216 | cartilage development | 0.010994 | 657/1280/3952/7040 |
| biological process | GO:0003081 | regulation of systemic arterial blood pressure by renin-angiotensin | 0.010994 | 183/1636 |
| biological process | GO:0031954 | positive regulation of protein autophosphorylation | 0.010994 | 805/1636 |
| biological process | GO:0032800 | receptor biosynthetic process | 0.010994 | 5465/9370 |
| biological process | GO:0043090 | amino acid import | 0.010994 | 183/477 |
| biological process | GO:0048753 | pigment granule organization | 0.010994 | 434/596 |
| biological process | GO:0060314 | regulation of ryanodine-sensitive calcium-release channel activity | 0.010994 | 805/845 |
| biological process | GO:1900739 | regulation of protein insertion into mitochondrial membrane involved in apoptotic signaling pathway | 0.010994 | 596/637 |
| biological process | GO:1900740 | positive regulation of protein insertion into mitochondrial membrane involved in apoptotic signaling pathway | 0.010994 | 596/637 |
| biological process | GO:1903579 | negative regulation of ATP metabolic process | 0.010994 | 142/5465 |
| biological process | GO:1903959 | regulation of anion transmembrane transport | 0.010994 | 183/477 |
| biological process | GO:0046364 | monosaccharide biosynthetic process | 0.011046 | 3952/5465/9370 |
| biological process | GO:0050764 | regulation of phagocytosis | 0.011046 | 5728/7040/9370 |
| biological process | GO:2001243 | negative regulation of intrinsic apoptotic signaling pathway | 0.011046 | 596/637/4780 |
| biological process | GO:0009161 | ribonucleoside monophosphate metabolic process | 0.011274 | 142/477/637/5465/7040 |
| biological process | GO:0071219 | cellular response to molecule of bacterial origin | 0.011451 | 671/1244/2950/7040 |
| biological process | GO:0010657 | muscle cell apoptotic process | 0.011588 | 183/4780/5728 |
| biological process | GO:0022600 | digestive system process | 0.011588 | 48/3952/5020 |
| biological process | GO:2001023 | regulation of response to drug | 0.011588 | 183/477/4780 |
| biological process | GO:1902175 | regulation of oxidative stress-induced intrinsic apoptotic signaling pathway | 0.011648 | 142/4780 |
| biological process | GO:0016051 | carbohydrate biosynthetic process | 0.011711 | 3952/5465/7040/9370 |
| biological process | GO:2001020 | regulation of response to DNA damage stimulus | 0.011711 | 142/596/637/5580 |
| biological process | GO:0046545 | development of primary female sexual characteristics | 0.01183 | 596/2189/3952 |
| biological process | GO:0009141 | nucleoside triphosphate metabolic process | 0.012209 | 142/477/637/5465/7040 |
| biological process | GO:0085029 | extracellular matrix assembly | 0.01238 | 183/7040 |
| biological process | GO:1900543 | negative regulation of purine nucleotide metabolic process | 0.01238 | 142/5465 |
| biological process | GO:0043583 | ear development | 0.012612 | 220/596/1280/7040 |
| biological process | GO:0046822 | regulation of nucleocytoplasmic transport | 0.012687 | 3952/5580/7040 |
| biological process | GO:0090100 | positive regulation of transmembrane receptor protein serine/threonine kinase signaling pathway | 0.012687 | 142/657/7040 |
| biological process | GO:2001237 | negative regulation of extrinsic apoptotic signaling pathway | 0.012687 | 596/1280/2950 |
| biological process | GO:0030198 | extracellular matrix organization | 0.012936 | 183/1280/1286/1462/7040 |
| biological process | GO:0006909 | phagocytosis | 0.013051 | 3952/5580/5728/7040/9370 |
| biological process | GO:0010800 | positive regulation of peptidyl-threonine phosphorylation | 0.013051 | 805/7040 |
| biological process | GO:0045980 | negative regulation of nucleotide metabolic process | 0.013051 | 142/5465 |
| biological process | GO:1901017 | negative regulation of potassium ion transmembrane transporter activity | 0.013051 | 845/5728 |
| biological process | GO:0006790 | sulfur compound metabolic process | 0.013427 | 1244/1462/2950/2952/4780 |
| biological process | GO:0043502 | regulation of muscle adaptation | 0.013574 | 142/183/5465 |
| biological process | GO:0033157 | regulation of intracellular protein transport | 0.01362 | 3952/5580/7040/9370 |
| biological process | GO:0009123 | nucleoside monophosphate metabolic process | 0.013805 | 142/477/637/5465/7040 |
| biological process | GO:0001844 | protein insertion into mitochondrial membrane involved in apoptotic signaling pathway | 0.013805 | 596/637 |
| biological process | GO:0071549 | cellular response to dexamethasone stimulus | 0.013805 | 1244/7040 |
| biological process | GO:0016101 | diterpenoid metabolic process | 0.014517 | 216/220/224 |
| biological process | GO:0007202 | activation of phospholipase C activity | 0.014517 | 183/1909 |
| biological process | GO:0019098 | reproductive behavior | 0.014517 | 5020/5728 |
| biological process | GO:0044319 | wound healing, spreading of cells | 0.014517 | 977/5728 |
| biological process | GO:0050849 | negative regulation of calcium-mediated signaling | 0.014517 | 805/845 |
| biological process | GO:0090505 | epiboly involved in wound healing | 0.014517 | 977/5728 |
| biological process | GO:1902230 | negative regulation of intrinsic apoptotic signaling pathway in response to DNA damage | 0.014517 | 596/637 |
| biological process | GO:1902895 | positive regulation of pri-miRNA transcription by RNA polymerase II | 0.014517 | 657/7040 |
| biological process | GO:0043279 | response to alkaloid | 0.015054 | 845/1393/5020 |
| biological process | GO:0051817 | modification of morphology or physiology of other organism involved in symbiotic interaction | 0.015054 | 805/820/7040 |
| biological process | GO:0042692 | muscle cell differentiation | 0.015114 | 183/596/845/5465/7040 |
| biological process | GO:0046685 | response to arsenic-containing substance | 0.015237 | 1244/5728 |
| biological process | GO:0055022 | negative regulation of cardiac muscle tissue growth | 0.015237 | 5465/5728 |
| biological process | GO:0061117 | negative regulation of heart growth | 0.015237 | 5465/5728 |
| biological process | GO:0061311 | cell surface receptor signaling pathway involved in heart development | 0.015237 | 657/7040 |
| biological process | GO:0090504 | epiboly | 0.015237 | 977/5728 |
| biological process | GO:0046620 | regulation of organ growth | 0.015243 | 657/5465/5728 |
| biological process | GO:0006469 | negative regulation of protein kinase activity | 0.015298 | 2950/5580/5728/9370 |
| biological process | GO:0009755 | hormone-mediated signaling pathway | 0.015298 | 142/434/1393/5465 |
| biological process | GO:0071216 | cellular response to biotic stimulus | 0.015504 | 671/1244/2950/7040 |
| biological process | GO:0030282 | bone mineralization | 0.015515 | 657/3952/7040 |
| biological process | GO:0046660 | female sex differentiation | 0.015874 | 596/2189/3952 |
| biological process | GO:0051048 | negative regulation of secretion | 0.015879 | 1393/3952/5020/9370 |
| biological process | GO:0010039 | response to iron ion | 0.015879 | 48/596 |
| biological process | GO:0042755 | eating behavior | 0.015879 | 3952/5020 |
| biological process | GO:0051204 | protein insertion into mitochondrial membrane | 0.015879 | 596/637 |
| biological process | GO:1901380 | negative regulation of potassium ion transmembrane transport | 0.015879 | 845/5728 |
| biological process | GO:0031348 | negative regulation of defense response | 0.015993 | 2950/5465/5580/9370 |
| biological process | GO:0021782 | glial cell development | 0.016066 | 2950/5728/7040 |
| biological process | GO:0010906 | regulation of glucose metabolic process | 0.016401 | 3952/5465/9370 |
| biological process | GO:0051153 | regulation of striated muscle cell differentiation | 0.016401 | 596/5465/7040 |
| biological process | GO:0032892 | positive regulation of organic acid transport | 0.016696 | 183/5020 |
| biological process | GO:0007173 | epidermal growth factor receptor signaling pathway | 0.017142 | 183/819/7040 |
| biological process | GO:0006721 | terpenoid metabolic process | 0.017482 | 216/220/224 |
| biological process | GO:0051954 | positive regulation of amine transport | 0.017482 | 183/5020 |
| biological process | GO:0071312 | cellular response to alkaloid | 0.017482 | 845/1393 |
| biological process | GO:1901030 | positive regulation of mitochondrial outer membrane permeabilization involved in apoptotic signaling pathway | 0.017482 | 596/637 |
| biological process | GO:1905332 | positive regulation of morphogenesis of an epithelium | 0.017482 | 183/7040 |
| biological process | GO:0022407 | regulation of cell-cell adhesion | 0.017482 | 3952/5465/5580/7040/9370 |
| biological process | GO:0019318 | hexose metabolic process | 0.018099 | 216/3952/5465/9370 |
| biological process | GO:0043500 | muscle adaptation | 0.018099 | 142/183/5465 |
| biological process | GO:1905477 | positive regulation of protein localization to membrane | 0.018099 | 596/637/7040 |
| biological process | GO:1901099 | negative regulation of signal transduction in absence of ligand | 0.018304 | 596/1280 |
| biological process | GO:2001240 | negative regulation of extrinsic apoptotic signaling pathway in absence of ligand | 0.018304 | 596/1280 |
| biological process | GO:0002062 | chondrocyte differentiation | 0.018424 | 657/1280/7040 |
| biological process | GO:0007517 | muscle organ development | 0.018584 | 596/657/5465/5728/7040 |
| biological process | GO:2000027 | regulation of animal organ morphogenesis | 0.018956 | 183/596/657/7040 |
| biological process | GO:0034405 | response to fluid shear stress | 0.019122 | 4780/7040 |
| biological process | GO:0043029 | T cell homeostasis | 0.019122 | 596/7040 |
| biological process | GO:0045923 | positive regulation of fatty acid metabolic process | 0.019122 | 5465/9370 |
| biological process | GO:0033673 | negative regulation of kinase activity | 0.019848 | 2950/5580/5728/9370 |
| biological process | GO:0048863 | stem cell differentiation | 0.019848 | 657/1636/1909/4780 |
| biological process | GO:0003298 | physiological muscle hypertrophy | 0.019848 | 183/5465 |
| biological process | GO:0003301 | physiological cardiac muscle hypertrophy | 0.019848 | 183/5465 |
| biological process | GO:0046326 | positive regulation of glucose import | 0.019848 | 4780/9370 |
| biological process | GO:0046329 | negative regulation of JNK cascade | 0.019848 | 259/2950 |
| biological process | GO:0050892 | intestinal absorption | 0.019848 | 48/3952 |
| biological process | GO:0061049 | cell growth involved in cardiac muscle cell development | 0.019848 | 183/5465 |
| biological process | GO:1903523 | negative regulation of blood circulation | 0.019848 | 477/3952 |
| biological process | GO:0009100 | glycoprotein metabolic process | 0.019897 | 28/596/1280/1462/3952 |
| biological process | GO:0010595 | positive regulation of endothelial cell migration | 0.020048 | 183/4780/7040 |
| biological process | GO:0043062 | extracellular structure organization | 0.020421 | 183/1280/1286/1462/7040 |
| biological process | GO:0034754 | cellular hormone metabolic process | 0.020421 | 216/220/1586 |
| biological process | GO:0051091 | positive regulation of DNA-binding transcription factor activity | 0.02057 | 183/847/5728/7040 |
| biological process | GO:0010661 | positive regulation of muscle cell apoptotic process | 0.020577 | 183/5728 |
| biological process | GO:0030501 | positive regulation of bone mineralization | 0.020577 | 657/7040 |
| biological process | GO:0045911 | positive regulation of DNA recombination | 0.020577 | 142/7040 |
| biological process | GO:1902229 | regulation of intrinsic apoptotic signaling pathway in response to DNA damage | 0.020577 | 596/637 |
| biological process | GO:0042476 | odontogenesis | 0.021308 | 657/5465/7040 |
| biological process | GO:0045598 | regulation of fat cell differentiation | 0.021308 | 3952/7040/9370 |
| biological process | GO:0045995 | regulation of embryonic development | 0.021308 | 657/4780/9370 |
| biological process | GO:0055007 | cardiac muscle cell differentiation | 0.021308 | 183/5465/7040 |
| biological process | GO:0001990 | regulation of systemic arterial blood pressure by hormone | 0.021308 | 183/1636 |
| biological process | GO:0007618 | mating | 0.021308 | 5020/5728 |
| biological process | GO:0044058 | regulation of digestive system process | 0.021308 | 3952/5020 |
| biological process | GO:0046621 | negative regulation of organ growth | 0.021308 | 5465/5728 |
| biological process | GO:0071548 | response to dexamethasone | 0.021308 | 1244/7040 |
| biological process | GO:0046883 | regulation of hormone secretion | 0.021496 | 183/1393/3952/9370 |
| biological process | GO:0031668 | cellular response to extracellular stimulus | 0.022036 | 596/2950/3952/4780 |
| biological process | GO:0042572 | retinol metabolic process | 0.022149 | 216/220 |
| biological process | GO:0043267 | negative regulation of potassium ion transport | 0.022149 | 845/5728 |
| biological process | GO:0050873 | brown fat cell differentiation | 0.022149 | 3952/9370 |
| biological process | GO:1903053 | regulation of extracellular matrix organization | 0.022149 | 183/7040 |
| biological process | GO:0048732 | gland development | 0.022184 | 220/596/657/5728/7040 |
| biological process | GO:0050804 | modulation of chemical synaptic transmission | 0.022579 | 183/477/5020/5728/9370 |
| biological process | GO:0099177 | regulation of trans-synaptic signaling | 0.022761 | 183/477/5020/5728/9370 |
| biological process | GO:0010470 | regulation of gastrulation | 0.022927 | 657/9370 |
| biological process | GO:0010613 | positive regulation of cardiac muscle hypertrophy | 0.022927 | 142/183 |
| biological process | GO:0010677 | negative regulation of cellular carbohydrate metabolic process | 0.022927 | 5465/9370 |
| biological process | GO:0042149 | cellular response to glucose starvation | 0.022927 | 596/4780 |
| biological process | GO:0019932 | second-messenger-mediated signaling | 0.022927 | 183/196/477/805/845 |
| biological process | GO:0043542 | endothelial cell migration | 0.022927 | 183/4780/5728/7040 |
| biological process | GO:0061448 | connective tissue development | 0.022927 | 657/1280/3952/7040 |
| biological process | GO:0031644 | regulation of neurological system process | 0.022927 | 183/5020/5728 |
| biological process | GO:0060048 | cardiac muscle contraction | 0.022927 | 477/805/845 |
| biological process | GO:0010863 | positive regulation of phospholipase C activity | 0.023607 | 183/1909 |
| biological process | GO:0014742 | positive regulation of muscle hypertrophy | 0.023607 | 142/183 |
| biological process | GO:0051602 | response to electrical stimulus | 0.023607 | 5020/5728 |
| biological process | GO:0055026 | negative regulation of cardiac muscle tissue development | 0.023607 | 5465/5728 |
| biological process | GO:0006720 | isoprenoid metabolic process | 0.023607 | 216/220/224 |
| biological process | GO:0007586 | digestion | 0.023607 | 48/3952/5020 |
| biological process | GO:0035304 | regulation of protein dephosphorylation | 0.023607 | 805/5580/7040 |
| biological process | GO:0042737 | drug catabolic process | 0.024044 | 847/1486/2329 |
| biological process | GO:0010828 | positive regulation of glucose transmembrane transport | 0.024559 | 4780/9370 |
| biological process | GO:1901028 | regulation of mitochondrial outer membrane permeabilization involved in apoptotic signaling pathway | 0.024559 | 596/637 |
| biological process | GO:0030522 | intracellular receptor signaling pathway | 0.024623 | 142/196/3952/5465 |
| biological process | GO:0010675 | regulation of cellular carbohydrate metabolic process | 0.024814 | 3952/5465/9370 |
| biological process | GO:0038127 | ERBB signaling pathway | 0.024814 | 183/819/7040 |
| biological process | GO:0030100 | regulation of endocytosis | 0.024817 | 977/5728/7040/9370 |
| biological process | GO:0032570 | response to progesterone | 0.025383 | 5020/7040 |
| biological process | GO:0090278 | negative regulation of peptide hormone secretion | 0.025383 | 1393/3952 |
| biological process | GO:1900274 | regulation of phospholipase C activity | 0.025383 | 183/1909 |
| biological process | GO:0031647 | regulation of protein stability | 0.025597 | 596/819/5580/5728 |
| biological process | GO:0015980 | energy derivation by oxidation of organic compounds | 0.025833 | 48/637/847/3952 |
| biological process | GO:0051348 | negative regulation of transferase activity | 0.025833 | 2950/5580/5728/9370 |
| biological process | GO:1904951 | positive regulation of establishment of protein localization | 0.025929 | 596/637/3952/5580/7040 |
| biological process | GO:0006865 | amino acid transport | 0.025929 | 183/477/3952 |
| biological process | GO:0044262 | cellular carbohydrate metabolic process | 0.025992 | 3952/5465/5728/9370 |
| biological process | GO:0051403 | stress-activated MAPK cascade | 0.025992 | 183/259/2950/3952 |
| biological process | GO:0010883 | regulation of lipid storage | 0.026172 | 3952/5465 |
| biological process | GO:0062012 | regulation of small molecule metabolic process | 0.02645 | 142/3952/5465/7040/9370 |
| biological process | GO:0010212 | response to ionizing radiation | 0.026725 | 142/596/7040 |
| biological process | GO:0046394 | carboxylic acid biosynthetic process | 0.026903 | 220/1462/5465/7040/9370 |
| biological process | GO:0010799 | regulation of peptidyl-threonine phosphorylation | 0.026903 | 805/7040 |
| biological process | GO:0014911 | positive regulation of smooth muscle cell migration | 0.026903 | 183/596 |
| biological process | GO:0070169 | positive regulation of biomineral tissue development | 0.026903 | 657/7040 |
| biological process | GO:0120163 | negative regulation of cold-induced thermogenesis | 0.026903 | 216/9370 |
| biological process | GO:2001239 | regulation of extrinsic apoptotic signaling pathway in absence of ligand | 0.026903 | 596/1280 |
| biological process | GO:0051592 | response to calcium ion | 0.026903 | 805/845/1393 |
| biological process | GO:0061041 | regulation of wound healing | 0.026903 | 4780/5580/5728 |
| biological process | GO:0002683 | negative regulation of immune system process | 0.026969 | 259/671/4780/7040/9370 |
| biological process | GO:0016053 | organic acid biosynthetic process | 0.026969 | 220/1462/5465/7040/9370 |
| biological process | GO:0010632 | regulation of epithelial cell migration | 0.027034 | 183/4780/5728/7040 |
| biological process | GO:0051092 | positive regulation of NF-kappaB transcription factor activity | 0.027204 | 183/847/7040 |
| biological process | GO:2000058 | regulation of ubiquitin-dependent protein catabolic process | 0.027204 | 819/4780/5728 |
| biological process | GO:0005996 | monosaccharide metabolic process | 0.027237 | 216/3952/5465/9370 |
| biological process | GO:0010862 | positive regulation of pathway-restricted SMAD protein phosphorylation | 0.027593 | 657/7040 |
| biological process | GO:0055010 | ventricular cardiac muscle tissue morphogenesis | 0.027593 | 657/7040 |
| biological process | GO:0060324 | face development | 0.027593 | 220/7040 |
| biological process | GO:1903727 | positive regulation of phospholipid metabolic process | 0.027593 | 5580/7040 |
| biological process | GO:0001818 | negative regulation of cytokine production | 0.028345 | 671/2950/7040/9370 |
| biological process | GO:0003044 | regulation of systemic arterial blood pressure mediated by a chemical signal | 0.028469 | 183/1636 |
| biological process | GO:0003254 | regulation of membrane depolarization | 0.028469 | 142/596 |
| biological process | GO:0043124 | negative regulation of I-kappaB kinase/NF-kappaB signaling | 0.028469 | 2950/9370 |
| biological process | GO:0051205 | protein insertion into membrane | 0.028469 | 596/637 |
| biological process | GO:1904707 | positive regulation of vascular smooth muscle cell proliferation | 0.028469 | 183/657 |
| biological process | GO:0034250 | positive regulation of cellular amide metabolic process | 0.028706 | 1153/4780/5580 |
| biological process | GO:0090316 | positive regulation of intracellular protein transport | 0.028706 | 3952/5580/7040 |
| biological process | GO:0050769 | positive regulation of neurogenesis | 0.02898 | 183/596/4780/5728/7040 |
| biological process | GO:0070372 | regulation of ERK1 and ERK2 cascade | 0.029223 | 2950/5728/7040/9370 |
| biological process | GO:0032873 | negative regulation of stress-activated MAPK cascade | 0.029223 | 259/2950 |
| biological process | GO:0033059 | cellular pigmentation | 0.029223 | 434/596 |
| biological process | GO:0070303 | negative regulation of stress-activated protein kinase signaling cascade | 0.029223 | 259/2950 |
| biological process | GO:0086009 | membrane repolarization | 0.029223 | 477/845 |
| biological process | GO:2000725 | regulation of cardiac muscle cell differentiation | 0.029223 | 5465/7040 |
| biological process | GO:0045216 | cell-cell junction organization | 0.029928 | 183/1636/7040 |
| biological process | GO:0031103 | axon regeneration | 0.030063 | 596/5728 |
| biological process | GO:0045599 | negative regulation of fat cell differentiation | 0.030063 | 7040/9370 |
| biological process | GO:0048146 | positive regulation of fibroblast proliferation | 0.030063 | 183/7040 |
| biological process | GO:0048641 | regulation of skeletal muscle tissue development | 0.030063 | 596/7040 |
| biological process | GO:0051452 | intracellular pH reduction | 0.030063 | 533/596 |
| biological process | GO:2001258 | negative regulation of cation channel activity | 0.030063 | 805/845 |
| biological process | GO:0003179 | heart valve morphogenesis | 0.031031 | 657/7040 |
| biological process | GO:0035176 | social behavior | 0.031031 | 5020/5728 |
| biological process | GO:0051703 | intraspecies interaction between organisms | 0.031031 | 5020/5728 |
| biological process | GO:2000677 | regulation of transcription regulatory region DNA binding | 0.031031 | 142/7040 |
| biological process | GO:0043312 | neutrophil degranulation | 0.031031 | 671/820/847/2950/5580 |
| biological process | GO:0006754 | ATP biosynthetic process | 0.031531 | 142/5465/7040 |
| biological process | GO:0002283 | neutrophil activation involved in immune response | 0.031734 | 671/820/847/2950/5580 |
| biological process | GO:0042113 | B cell activation | 0.031885 | 196/596/5580/7040 |
| biological process | GO:0010043 | response to zinc ion | 0.031885 | 142/5728 |
| biological process | GO:0045851 | pH reduction | 0.031885 | 533/596 |
| biological process | GO:1902475 | L-alpha-amino acid transmembrane transport | 0.031885 | 183/477 |
| biological process | GO:0046879 | hormone secretion | 0.032422 | 183/1393/3952/9370 |
| biological process | GO:0050994 | regulation of lipid catabolic process | 0.032919 | 5465/5580 |
| biological process | GO:0097345 | mitochondrial outer membrane permeabilization | 0.032919 | 596/637 |
| biological process | GO:0035821 | modification of morphology or physiology of other organism | 0.033272 | 805/820/7040 |
| biological process | GO:0031098 | stress-activated protein kinase signaling cascade | 0.033272 | 183/259/2950/3952 |
| biological process | GO:0070588 | calcium ion transmembrane transport | 0.033272 | 477/805/845/7040 |
| biological process | GO:0001541 | ovarian follicle development | 0.033711 | 596/2189 |
| biological process | GO:0002011 | morphogenesis of an epithelial sheet | 0.033711 | 977/5728 |
| biological process | GO:0003229 | ventricular cardiac muscle tissue development | 0.033711 | 657/7040 |
| biological process | GO:0042220 | response to cocaine | 0.033711 | 1393/5020 |
| biological process | GO:0061098 | positive regulation of protein tyrosine kinase activity | 0.033711 | 183/1636 |
| biological process | GO:0042119 | neutrophil activation | 0.033711 | 671/820/847/2950/5580 |
| biological process | GO:0035051 | cardiocyte differentiation | 0.034557 | 183/5465/7040 |
| biological process | GO:0010332 | response to gamma radiation | 0.034681 | 142/596 |
| biological process | GO:0032615 | interleukin-12 production | 0.034681 | 3952/5580 |
| biological process | GO:0060688 | regulation of morphogenesis of a branching structure | 0.034681 | 183/7040 |
| biological process | GO:0051302 | regulation of cell division | 0.03493 | 805/847/7040 |
| biological process | GO:0018209 | peptidyl-serine modification | 0.035236 | 142/596/5580/7040 |
| biological process | GO:0060562 | epithelial tube morphogenesis | 0.035236 | 183/596/1909/7040 |
| biological process | GO:0048008 | platelet-derived growth factor receptor signaling pathway | 0.03559 | 5728/9370 |
| biological process | GO:0060043 | regulation of cardiac muscle cell proliferation | 0.03559 | 657/5728 |
| biological process | GO:2000272 | negative regulation of signaling receptor activity | 0.03559 | 1393/5728 |
| biological process | GO:0006022 | aminoglycan metabolic process | 0.035776 | 1462/1486/7040 |
| biological process | GO:0009206 | purine ribonucleoside triphosphate biosynthetic process | 0.036251 | 142/5465/7040 |
| biological process | GO:0010634 | positive regulation of epithelial cell migration | 0.036251 | 183/4780/7040 |
| biological process | GO:0031663 | lipopolysaccharide-mediated signaling pathway | 0.036503 | 671/7040 |
| biological process | GO:0035904 | aorta development | 0.036503 | 657/3952 |
| biological process | GO:1903749 | positive regulation of establishment of protein localization to mitochondrion | 0.036503 | 596/637 |
| biological process | GO:0009145 | purine nucleoside triphosphate biosynthetic process | 0.03663 | 142/5465/7040 |
| biological process | GO:0010469 | regulation of signaling receptor activity | 0.03716 | 1393/5580/5728 |
| biological process | GO:0001836 | release of cytochrome c from mitochondria | 0.037368 | 596/637 |
| biological process | GO:0010518 | positive regulation of phospholipase activity | 0.037368 | 183/1909 |
| biological process | GO:0031102 | neuron projection regeneration | 0.037368 | 596/5728 |
| biological process | GO:0043388 | positive regulation of DNA binding | 0.037368 | 142/7040 |
| biological process | GO:0097755 | positive regulation of blood vessel diameter | 0.037368 | 183/3952 |
| biological process | GO:0006941 | striated muscle contraction | 0.037393 | 477/805/845 |
| biological process | GO:0032496 | response to lipopolysaccharide | 0.037393 | 671/1244/2950/7040 |
| biological process | GO:0008202 | steroid metabolic process | 0.037729 | 183/847/1586/3952 |
| biological process | GO:0090342 | regulation of cell aging | 0.038337 | 657/5728 |
| biological process | GO:1902110 | positive regulation of mitochondrial membrane permeability involved in apoptotic process | 0.038337 | 596/637 |
| biological process | GO:0009201 | ribonucleoside triphosphate biosynthetic process | 0.038953 | 142/5465/7040 |
| biological process | GO:0003170 | heart valve development | 0.039135 | 657/7040 |
| biological process | GO:0006027 | glycosaminoglycan catabolic process | 0.039135 | 1462/7040 |
| biological process | GO:0010803 | regulation of tumor necrosis factor-mediated signaling pathway | 0.039135 | 2950/9370 |
| biological process | GO:0030888 | regulation of B cell proliferation | 0.039135 | 196/596 |
| biological process | GO:1905953 | negative regulation of lipid localization | 0.039135 | 3952/5465 |
| biological process | GO:2001244 | positive regulation of intrinsic apoptotic signaling pathway | 0.039135 | 596/637 |
| biological process | GO:0050821 | protein stabilization | 0.039135 | 819/5580/5728 |
| biological process | GO:2001257 | regulation of cation channel activity | 0.039135 | 805/845/1393 |
| biological process | GO:0043405 | regulation of MAP kinase activity | 0.039471 | 2950/5580/7040/9370 |
| biological process | GO:0051099 | positive regulation of binding | 0.039575 | 142/1636/7040 |
| biological process | GO:1903034 | regulation of response to wounding | 0.039575 | 4780/5580/5728 |
| biological process | GO:1905330 | regulation of morphogenesis of an epithelium | 0.039767 | 183/5728/7040 |
| biological process | GO:0002260 | lymphocyte homeostasis | 0.039767 | 596/7040 |
| biological process | GO:0007585 | respiratory gaseous exchange | 0.039767 | 477/1909 |
| biological process | GO:0032922 | circadian regulation of gene expression | 0.039767 | 196/5465 |
| biological process | GO:0050433 | regulation of catecholamine secretion | 0.039767 | 183/5020 |
| biological process | GO:0055025 | positive regulation of cardiac muscle tissue development | 0.039767 | 657/7040 |
| biological process | GO:0060393 | regulation of pathway-restricted SMAD protein phosphorylation | 0.039767 | 657/7040 |
| biological process | GO:1902686 | mitochondrial outer membrane permeabilization involved in programmed cell death | 0.039767 | 596/637 |
| biological process | GO:0051147 | regulation of muscle cell differentiation | 0.040303 | 596/5465/7040 |
| biological process | GO:0007588 | excretion | 0.040793 | 183/5020 |
| biological process | GO:0032371 | regulation of sterol transport | 0.040793 | 3952/9370 |
| biological process | GO:0032374 | regulation of cholesterol transport | 0.040793 | 3952/9370 |
| biological process | GO:0002237 | response to molecule of bacterial origin | 0.040987 | 671/1244/2950/7040 |
| biological process | GO:0006913 | nucleocytoplasmic transport | 0.040987 | 183/3952/5580/7040 |
| biological process | GO:0072330 | monocarboxylic acid biosynthetic process | 0.040987 | 220/1462/5465/9370 |
| biological process | GO:0009127 | purine nucleoside monophosphate biosynthetic process | 0.041088 | 142/5465/7040 |
| biological process | GO:0009168 | purine ribonucleoside monophosphate biosynthetic process | 0.041088 | 142/5465/7040 |
| biological process | GO:0042098 | T cell proliferation | 0.041453 | 977/3952/7040 |
| biological process | GO:0048639 | positive regulation of developmental growth | 0.041453 | 596/657/3952 |
| biological process | GO:0050864 | regulation of B cell activation | 0.041453 | 196/596/7040 |
| biological process | GO:2000045 | regulation of G1/S transition of mitotic cell cycle | 0.041453 | 596/637/5728 |
| biological process | GO:0035794 | positive regulation of mitochondrial membrane permeability | 0.041453 | 596/637 |
| biological process | GO:0050432 | catecholamine secretion | 0.041453 | 183/5020 |
| biological process | GO:0051169 | nuclear transport | 0.041728 | 183/3952/5580/7040 |
| biological process | GO:0070997 | neuron death | 0.042376 | 142/596/637/5465 |
| biological process | GO:0006940 | regulation of smooth muscle contraction | 0.042376 | 477/5020 |
| biological process | GO:0051187 | cofactor catabolic process | 0.042376 | 259/847 |
| biological process | GO:0060389 | pathway-restricted SMAD protein phosphorylation | 0.042376 | 657/7040 |
| biological process | GO:1901016 | regulation of potassium ion transmembrane transporter activity | 0.042376 | 845/5728 |
| biological process | GO:1905475 | regulation of protein localization to membrane | 0.042833 | 596/637/7040 |
| biological process | GO:0009142 | nucleoside triphosphate biosynthetic process | 0.043248 | 142/5465/7040 |
| biological process | GO:0010631 | epithelial cell migration | 0.043248 | 183/4780/5728/7040 |
| biological process | GO:0051260 | protein homooligomerization | 0.043248 | 220/637/847/9370 |
| biological process | GO:1902108 | regulation of mitochondrial membrane permeability involved in apoptotic process | 0.043248 | 596/637 |
| biological process | GO:1905207 | regulation of cardiocyte differentiation | 0.043248 | 5465/7040 |
| biological process | GO:1905710 | positive regulation of membrane permeability | 0.043248 | 596/637 |
| biological process | GO:0071248 | cellular response to metal ion | 0.044279 | 142/1393/4780 |
| biological process | GO:0090132 | epithelium migration | 0.044279 | 183/4780/5728/7040 |
| biological process | GO:0015807 | L-amino acid transport | 0.044285 | 183/477 |
| biological process | GO:0060038 | cardiac muscle cell proliferation | 0.044285 | 657/5728 |
| biological process | GO:0048839 | inner ear development | 0.045342 | 220/1280/7040 |
| biological process | GO:0019915 | lipid storage | 0.045439 | 3952/5465 |
| biological process | GO:0045333 | cellular respiration | 0.045862 | 48/637/847 |
| biological process | GO:0090130 | tissue migration | 0.046498 | 183/4780/5728/7040 |
| biological process | GO:0055008 | cardiac muscle tissue morphogenesis | 0.046547 | 657/7040 |
| biological process | GO:0031331 | positive regulation of cellular catabolic process | 0.046816 | 4780/5465/5580/5728 |
| biological process | GO:0045861 | negative regulation of proteolysis | 0.047602 | 183/259/819/1469 |
| biological process | GO:0010517 | regulation of phospholipase activity | 0.047602 | 183/1909 |
| biological process | GO:0072091 | regulation of stem cell proliferation | 0.047602 | 1636/7040 |
| biological process | GO:0009156 | ribonucleoside monophosphate biosynthetic process | 0.047967 | 142/5465/7040 |
| biological process | GO:0009749 | response to glucose | 0.047967 | 5728/7040/9370 |
| biological process | GO:0007626 | locomotory behavior | 0.048499 | 220/477/5728 |
| biological process | GO:0046890 | regulation of lipid biosynthetic process | 0.048499 | 3952/5580/9370 |
| biological process | GO:0007179 | transforming growth factor beta receptor signaling pathway | 0.048974 | 142/657/7040 |
| biological process | GO:0016052 | carbohydrate catabolic process | 0.048974 | 216/1486/5465 |
| biological process | GO:0050866 | negative regulation of cell activation | 0.048974 | 671/5580/7040 |
| biological process | GO:0038034 | signal transduction in absence of ligand | 0.049368 | 596/1280 |
| biological process | GO:0050805 | negative regulation of synaptic transmission | 0.049368 | 5728/9370 |
| biological process | GO:0051155 | positive regulation of striated muscle cell differentiation | 0.049368 | 596/7040 |
| biological process | GO:0060193 | positive regulation of lipase activity | 0.049368 | 183/1909 |
| biological process | GO:0061035 | regulation of cartilage development | 0.049368 | 3952/7040 |
| biological process | GO:0097192 | extrinsic apoptotic signaling pathway in absence of ligand | 0.049368 | 596/1280 |
| biological process | GO:1903747 | regulation of establishment of protein localization to mitochondrion | 0.049368 | 596/637 |
| cellular component | GO:0062023 | collagen-containing extracellular matrix | 0.00083 | 183/259/977/1280/1286/1462/7040/9370 |
| cellular component | GO:0034774 | secretory granule lumen | 0.006188 | 671/820/847/2950/5580/7040 |
| cellular component | GO:0060205 | cytoplasmic vesicle lumen | 0.006188 | 671/820/847/2950/5580/7040 |
| cellular component | GO:0031983 | vesicle lumen | 0.006188 | 671/820/847/2950/5580/7040 |
| cellular component | GO:0005775 | vacuolar lumen | 0.024162 | 53/671/1462/5580 |
| cellular component | GO:0098644 | complex of collagen trimers | 0.024162 | 1280/1286 |
| cellular component | GO:0005581 | collagen trimer | 0.026644 | 1280/1286/9370 |
| cellular component | GO:0005604 | basement membrane | 0.030033 | 977/1280/1286 |
| molecular function | GO:0004029 | aldehyde dehydrogenase (NAD) activity | 0.00214 | 216/220/224 |
| molecular function | GO:0016620 | oxidoreductase activity, acting on the aldehyde or oxo group of donors, NAD or NADP as acceptor | 0.007862 | 216/220/224 |
| molecular function | GO:0050662 | coenzyme binding | 0.007862 | 36/142/216/220/847/2329 |
| molecular function | GO:0016248 | channel inhibitor activity | 0.007862 | 259/596/805 |
| molecular function | GO:0042562 | hormone binding | 0.007862 | 216/220/477/1393 |
| molecular function | GO:0001221 | transcription cofactor binding | 0.007862 | 196/4780/5465 |
| molecular function | GO:0016903 | oxidoreductase activity, acting on the aldehyde or oxo group of donors | 0.007862 | 216/220/224 |
| molecular function | GO:0005179 | hormone activity | 0.010025 | 183/3952/5020/9370 |
| molecular function | GO:0004601 | peroxidase activity | 0.010025 | 847/2950/2952 |
| molecular function | GO:0019855 | calcium channel inhibitor activity | 0.010025 | 259/805 |
| molecular function | GO:0016684 | oxidoreductase activity, acting on peroxide as acceptor | 0.01005 | 847/2950/2952 |
| molecular function | GO:0051287 | NAD binding | 0.01005 | 142/216/220 |
| molecular function | GO:0005201 | extracellular matrix structural constituent | 0.019462 | 1280/1286/1462/9370 |
| molecular function | GO:0004602 | glutathione peroxidase activity | 0.026872 | 2950/2952 |
| molecular function | GO:0016209 | antioxidant activity | 0.02804 | 847/2950/2952 |
| molecular function | GO:0001223 | transcription coactivator binding | 0.02823 | 196/5465 |
| molecular function | GO:0004364 | glutathione transferase activity | 0.036607 | 2950/2952 |
| molecular function | GO:0043539 | protein serine/threonine kinase activator activity | 0.039853 | 805/7040 |
| molecular function | GO:0047485 | protein N-terminus binding | 0.043474 | 142/805/7040 |
| molecular function | GO:0042626 | ATPase activity, coupled to transmembrane movement of substances | 0.045739 | 477/533/1244 |
| molecular function | GO:0043492 | ATPase activity, coupled to movement of substances | 0.045739 | 477/533/1244 |
| molecular function | GO:0001530 | lipopolysaccharide binding | 0.047294 | 671/820 |
| molecular function | GO:0015405 | P-P-bond-hydrolysis-driven transmembrane transporter activity | 0.048359 | 477/533/1244 |
| molecular function | GO:0008200 | ion channel inhibitor activity | 0.048359 | 259/805 |
| molecular function | GO:0015399 | primary active transmembrane transporter activity | 0.048519 | 477/533/1244 |
| KEGG | hsa01524 | Platinum drug resistance | 0.004931 | 596/637/1244/2950/2952 |
| KEGG | hsa05418 | Fluid shear stress and atherosclerosis | 0.005046 | 596/657/805/2950/2952/4780 |
| KEGG | hsa04933 | AGE-RAGE signaling pathway in diabetic complications | 0.007472 | 183/596/1286/5580/7040 |
| KEGG | hsa05152 | Tuberculosis | 0.010513 | 533/596/637/805/820/7040 |
| KEGG | hsa04924 | Renin secretion | 0.012916 | 183/805/1636/1909 |
| KEGG | hsa04932 | Non-alcoholic fatty liver disease | 0.0245 | 637/3952/5465/7040/9370 |
| KEGG | hsa04970 | Salivary secretion | 0.02861 | 477/805/820/1469 |
| KEGG | hsa05225 | Hepatocellular carcinoma | 0.030565 | 2950/2952/4780/5728/7040 |
| KEGG | hsa04931 | Insulin resistance | 0.038723 | 183/5465/5580/5728 |
| KEGG | hsa04024 | cAMP signaling pathway | 0.067395 | 477/805/1909/5020/5465 |
| KEGG | hsa04270 | Vascular smooth muscle contraction | 0.067395 | 183/805/1909/5580 |
| KEGG | hsa04920 | Adipocytokine signaling pathway | 0.069051 | 3952/5465/9370 |
| KEGG | hsa00982 | Drug metabolism - cytochrome P450 | 0.069051 | 2329/2950/2952 |
| KEGG | hsa04115 | p53 signaling pathway | 0.069051 | 596/637/5728 |
| KEGG | hsa04614 | Renin-angiotensin system | 0.069051 | 183/1636 |
| KEGG | hsa04261 | Adrenergic signaling in cardiomyocytes | 0.07095 | 183/477/596/805 |
| KEGG | hsa00630 | Glyoxylate and dicarboxylate metabolism | 0.098722 | 48/847 |
| KEGG | hsa05410 | Hypertrophic cardiomyopathy | 0.098722 | 183/1636/7040 |
| KEGG | hsa05222 | Small cell lung cancer | 0.098722 | 596/1286/5728 |
| KEGG | hsa04215 | Apoptosis - multiple species | 0.098722 | 596/637 |
| KEGG | hsa05215 | Prostate cancer | 0.102749 | 596/2950/5728 |
| KEGG | hsa04925 | Aldosterone synthesis and secretion | 0.102749 | 183/477/805 |
| KEGG | hsa04974 | Protein digestion and absorption | 0.112355 | 477/1280/1286 |
| KEGG | hsa04510 | Focal adhesion | 0.128667 | 596/1280/1286/5728 |
| KEGG | hsa00380 | Tryptophan metabolism | 0.132901 | 224/847 |
| KEGG | hsa00071 | Fatty acid degradation | 0.133608 | 36/224 |
| KEGG | hsa04071 | Sphingolipid signaling pathway | 0.135357 | 596/637/5728 |
| KEGG | hsa04722 | Neurotrophin signaling pathway | 0.135357 | 596/805/5580 |
| KEGG | hsa04930 | Type II diabetes mellitus | 0.136026 | 5580/9370 |
| KEGG | hsa00280 | Valine, leucine and isoleucine degradation | 0.142422 | 36/224 |
| KEGG | hsa04068 | FoxO signaling pathway | 0.156984 | 847/5728/7040 |
| KEGG | hsa04210 | Apoptosis | 0.163612 | 142/596/637 |
| KEGG | hsa04140 | Autophagy - animal | 0.163612 | 596/5580/5728 |
| KEGG | hsa04915 | Estrogen signaling pathway | 0.163612 | 596/805/5580 |
| KEGG | hsa00480 | Glutathione metabolism | 0.167985 | 2950/2952 |
| KEGG | hsa04934 | Cushing syndrome | 0.199541 | 183/196/1586 |
| KEGG | hsa04927 | Cortisol synthesis and secretion | 0.199541 | 183/1586 |
| KEGG | hsa04218 | Cellular senescence | 0.199541 | 805/5728/7040 |
| KEGG | hsa04217 | Necroptosis | 0.202804 | 142/596/637 |
| KEGG | hsa00830 | Retinol metabolism | 0.202804 | 216/220 |
| KEGG | hsa05161 | Hepatitis B | 0.203124 | 596/637/7040 |
| KEGG | hsa04022 | cGMP-PKG signaling pathway | 0.213725 | 477/805/1909 |
| KEGG | hsa05214 | Glioma | 0.220061 | 805/5728 |
| KEGG | hsa03320 | PPAR signaling pathway | 0.220061 | 5465/9370 |
| KEGG | hsa04971 | Gastric acid secretion | 0.220061 | 477/805 |
| KEGG | hsa00980 | Metabolism of xenobiotics by cytochrome P450 | 0.222861 | 2950/2952 |
| KEGG | hsa01521 | EGFR tyrosine kinase inhibitor resistance | 0.222861 | 596/5728 |
| KEGG | hsa00983 | Drug metabolism - other enzymes | 0.222861 | 2950/2952 |
| KEGG | hsa04621 | NOD-like receptor signaling pathway | 0.222861 | 596/820/5580 |
| KEGG | hsa05204 | Chemical carcinogenesis | 0.231466 | 2950/2952 |
| KEGG | hsa05210 | Colorectal cancer | 0.241506 | 596/7040 |
| KEGG | hsa04260 | Cardiac muscle contraction | 0.241695 | 477/845 |
| KEGG | hsa04512 | ECM-receptor interaction | 0.241909 | 1280/1286 |
| KEGG | hsa04211 | Longevity regulating pathway | 0.242146 | 847/9370 |
| KEGG | hsa04976 | Bile secretion | 0.242405 | 477/1244 |
| KEGG | hsa04912 | GnRH signaling pathway | 0.243112 | 805/5580 |
| KEGG | hsa05323 | Rheumatoid arthritis | 0.243112 | 533/7040 |
| KEGG | hsa04350 | TGF-beta signaling pathway | 0.243112 | 657/7040 |
| KEGG | hsa05165 | Human papillomavirus infection | 0.243112 | 533/1280/1286/5728 |
| KEGG | hsa05414 | Dilated cardiomyopathy | 0.243112 | 183/7040 |
| KEGG | hsa04070 | Phosphatidylinositol signaling system | 0.243112 | 805/5728 |
| KEGG | hsa04750 | Inflammatory mediator regulation of TRP channels | 0.243112 | 805/5580 |
| KEGG | hsa04080 | Neuroactive ligand-receptor interaction | 0.243112 | 183/1909/3952/5020 |
| KEGG | hsa05170 | Human immunodeficiency virus 1 infection | 0.243112 | 596/637/805 |
| KEGG | hsa04916 | Melanogenesis | 0.243112 | 434/805 |
| KEGG | hsa01210 | 2-Oxocarboxylic acid metabolism | 0.243112 | 48 |
| KEGG | hsa05142 | Chagas disease | 0.243112 | 1636/7040 |
| KEGG | hsa05146 | Amoebiasis | 0.243112 | 1286/7040 |
| KEGG | hsa04064 | NF-kappa B signaling pathway | 0.244077 | 142/596 |
| KEGG | hsa04625 | C-type lectin receptor signaling pathway | 0.244077 | 805/5580 |
| KEGG | hsa04151 | PI3K-Akt signaling pathway | 0.245014 | 596/1280/1286/5728 |
| KEGG | hsa04659 | Th17 cell differentiation | 0.245014 | 196/7040 |
| KEGG | hsa04922 | Glucagon signaling pathway | 0.245014 | 805/5465 |
| KEGG | hsa00770 | Pantothenate and CoA biosynthesis | 0.245014 | 224 |
| KEGG | hsa00340 | Histidine metabolism | 0.252657 | 224 |
| KEGG | hsa05145 | Toxoplasmosis | 0.254648 | 596/7040 |
| KEGG | hsa04964 | Proximal tubule bicarbonate reclamation | 0.256669 | 477 |
| KEGG | hsa04020 | Calcium signaling pathway | 0.267158 | 805/845/1909 |
| KEGG | hsa01200 | Carbon metabolism | 0.267158 | 48/847 |
| KEGG | hsa04152 | AMPK signaling pathway | 0.271231 | 3952/9370 |
| KEGG | hsa00601 | Glycosphingolipid biosynthesis - lacto and neolacto series | 0.283719 | 28 |
| KEGG | hsa04744 | Phototransduction | 0.289951 | 805 |
| KEGG | hsa04142 | Lysosome | 0.290507 | 53/533 |
| KEGG | hsa04926 | Relaxin signaling pathway | 0.290692 | 1286/7040 |
| KEGG | hsa00020 | Citrate cycle (TCA cycle) | 0.291424 | 48 |
| KEGG | hsa00053 | Ascorbate and aldarate metabolism | 0.291424 | 224 |
| KEGG | hsa00410 | beta-Alanine metabolism | 0.291424 | 224 |
| KEGG | hsa01523 | Antifolate resistance | 0.297013 | 1244 |
| KEGG | hsa05162 | Measles | 0.307692 | 596/637 |
| KEGG | hsa03410 | Base excision repair | 0.307692 | 142 |
| KEGG | hsa05226 | Gastric cancer | 0.334321 | 596/7040 |
| KEGG | hsa04960 | Aldosterone-regulated sodium reabsorption | 0.334321 | 477 |
| KEGG | hsa00620 | Pyruvate metabolism | 0.343351 | 224 |
| KEGG | hsa04921 | Oxytocin signaling pathway | 0.343351 | 805/5020 |
| KEGG | hsa04060 | Cytokine-cytokine receptor interaction | 0.343351 | 657/3952/7040 |
| KEGG | hsa04390 | Hippo signaling pathway | 0.343351 | 657/7040 |
| KEGG | hsa05160 | Hepatitis C | 0.343351 | 637/5465 |
| KEGG | hsa04630 | JAK-STAT signaling pathway | 0.356563 | 596/3952 |
| KEGG | hsa02010 | ABC transporters | 0.370829 | 1244 |
| KEGG | hsa05022 | Pathways of neurodegeneration - multiple diseases | 0.374166 | 596/637/805/847 |
| KEGG | hsa04141 | Protein processing in endoplasmic reticulum | 0.374166 | 596/4780 |
| KEGG | hsa04973 | Carbohydrate digestion and absorption | 0.374166 | 477 |
| KEGG | hsa04672 | Intestinal immune network for IgA production | 0.379892 | 7040 |
| KEGG | hsa04340 | Hedgehog signaling pathway | 0.379892 | 596 |
| KEGG | hsa05110 | Vibrio cholerae infection | 0.379892 | 533 |
| KEGG | hsa05144 | Malaria | 0.379892 | 7040 |
| KEGG | hsa00330 | Arginine and proline metabolism | 0.379892 | 224 |
| KEGG | hsa04913 | Ovarian steroidogenesis | 0.379892 | 1586 |
| KEGG | hsa04961 | Endocrine and other factor-regulated calcium reabsorption | 0.389352 | 477 |
| KEGG | hsa03460 | Fanconi anemia pathway | 0.392179 | 2189 |
| KEGG | hsa01212 | Fatty acid metabolism | 0.404446 | 36 |
| KEGG | hsa05167 | Kaposi sarcoma-associated herpesvirus infection | 0.404446 | 637/805 |
| KEGG | hsa05213 | Endometrial cancer | 0.406259 | 5728 |
| KEGG | hsa04978 | Mineral absorption | 0.407369 | 477 |
| KEGG | hsa05416 | Viral myocarditis | 0.407369 | 637 |
| KEGG | hsa00140 | Steroid hormone biosynthesis | 0.407369 | 1586 |
| KEGG | hsa00561 | Glycerolipid metabolism | 0.407369 | 224 |
| KEGG | hsa05169 | Epstein-Barr virus infection | 0.407369 | 596/637 |
| KEGG | hsa04213 | Longevity regulating pathway - multiple species | 0.407369 | 847 |
| KEGG | hsa05014 | Amyotrophic lateral sclerosis | 0.407369 | 596/637/847 |
| KEGG | hsa00310 | Lysine degradation | 0.407369 | 224 |
| KEGG | hsa05321 | Inflammatory bowel disease | 0.414939 | 7040 |
| KEGG | hsa00010 | Glycolysis / Gluconeogenesis | 0.416077 | 224 |
| KEGG | hsa04720 | Long-term potentiation | 0.416077 | 805 |
| KEGG | hsa05031 | Amphetamine addiction | 0.416077 | 805 |
| KEGG | hsa05211 | Renal cell carcinoma | 0.416077 | 7040 |
| KEGG | hsa04917 | Prolactin signaling pathway | 0.416077 | 1586 |
| KEGG | hsa05120 | Epithelial cell signaling in Helicobacter pylori infection | 0.416077 | 533 |
| KEGG | hsa05230 | Central carbon metabolism in cancer | 0.416077 | 5728 |
| KEGG | hsa05166 | Human T-cell leukemia virus 1 infection | 0.416077 | 5728/7040 |
| KEGG | hsa05218 | Melanoma | 0.418221 | 5728 |
| KEGG | hsa00562 | Inositol phosphate metabolism | 0.418221 | 5728 |
| KEGG | hsa05163 | Human cytomegalovirus infection | 0.418221 | 637/805 |
| KEGG | hsa01230 | Biosynthesis of amino acids | 0.418221 | 48 |
| KEGG | hsa04918 | Thyroid hormone synthesis | 0.418221 | 477 |
| KEGG | hsa05133 | Pertussis | 0.418221 | 805 |
| KEGG | hsa05212 | Pancreatic cancer | 0.418221 | 7040 |
| KEGG | hsa05220 | Chronic myeloid leukemia | 0.418221 | 7040 |
| KEGG | hsa05140 | Leishmaniasis | 0.419716 | 7040 |
| KEGG | hsa04721 | Synaptic vesicle cycle | 0.421168 | 533 |
| KEGG | hsa04146 | Peroxisome | 0.439951 | 847 |
| KEGG | hsa05131 | Shigellosis | 0.444764 | 596/5580 |
| KEGG | hsa04911 | Insulin secretion | 0.445515 | 477 |
| KEGG | hsa05012 | Parkinson disease | 0.445515 | 805/4780 |
| KEGG | hsa05235 | PD-L1 expression and PD-1 checkpoint pathway in cancer | 0.452545 | 5728 |
| KEGG | hsa05150 | Staphylococcus aureus infection | 0.474591 | 820 |
| KEGG | hsa04666 | Fc gamma R-mediated phagocytosis | 0.474591 | 5580 |
| KEGG | hsa04713 | Circadian entrainment | 0.474591 | 805 |
| KEGG | hsa01522 | Endocrine resistance | 0.475198 | 596 |
| KEGG | hsa04972 | Pancreatic secretion | 0.486911 | 477 |
| KEGG | hsa04928 | Parathyroid hormone synthesis, secretion and action | 0.498183 | 596 |
| KEGG | hsa04066 | HIF-1 signaling pathway | 0.505522 | 596 |
| KEGG | hsa04725 | Cholinergic synapse | 0.516042 | 596 |
| KEGG | hsa04919 | Thyroid hormone signaling pathway | 0.539364 | 477 |
| KEGG | hsa05206 | MicroRNAs in cancer | 0.54016 | 596/5728 |
| KEGG | hsa04110 | Cell cycle | 0.54206 | 7040 |
| KEGG | hsa04380 | Osteoclast differentiation | 0.550705 | 7040 |
| KEGG | hsa04114 | Oocyte meiosis | 0.550705 | 805 |
| KEGG | hsa04650 | Natural killer cell mediated cytotoxicity | 0.552352 | 637 |
| KEGG | hsa04728 | Dopaminergic synapse | 0.552352 | 805 |
| KEGG | hsa00190 | Oxidative phosphorylation | 0.552352 | 533 |
| KEGG | hsa04371 | Apelin signaling pathway | 0.557107 | 805 |
| KEGG | hsa04910 | Insulin signaling pathway | 0.557107 | 805 |
| KEGG | hsa04550 | Signaling pathways regulating pluripotency of stem cells | 0.570486 | 657 |
| KEGG | hsa05224 | Breast cancer | 0.576233 | 5728 |
| KEGG | hsa04072 | Phospholipase D signaling pathway | 0.576233 | 183 |
| KEGG | hsa04514 | Cell adhesion molecules | 0.576233 | 1462 |
| KEGG | hsa04145 | Phagosome | 0.580578 | 533 |
| KEGG | hsa04150 | mTOR signaling pathway | 0.583818 | 5728 |
| KEGG | hsa01240 | Biosynthesis of cofactors | 0.583818 | 224 |
| KEGG | hsa05010 | Alzheimer disease | 0.589954 | 637/805 |
| KEGG | hsa05164 | Influenza A | 0.61265 | 637 |
| KEGG | hsa05034 | Alcoholism | 0.6441 | 805 |
| KEGG | hsa04062 | Chemokine signaling pathway | 0.650717 | 5580 |
| KEGG | hsa05205 | Proteoglycans in cancer | 0.672514 | 7040 |
| KEGG | hsa04015 | Rap1 signaling pathway | 0.678023 | 805 |
| KEGG | hsa04014 | Ras signaling pathway | 0.708379 | 805 |
| KEGG | hsa05171 | Coronavirus disease - COVID-19 | 0.708379 | 1636 |
| KEGG | hsa05132 | Salmonella infection | 0.727854 | 596 |
| KEGG | hsa05168 | Herpes simplex virus 1 infection | 0.727854 | 596/637 |
| KEGG | hsa05020 | Prion disease | 0.756197 | 5580 |
| KEGG | hsa04010 | MAPK signaling pathway | 0.777988 | 7040 |
| KEGG | hsa04740 | Olfactory transduction | 0.897902 | 805 |
